# The roles of *MTRR* and *MTHFR* gene polymorphisms in congenital heart diseases: a meta-analysis

**DOI:** 10.1042/BSR20181160

**Published:** 2018-12-07

**Authors:** Aiping Xu, Weiping Wang, Xiaolei Jiang

**Affiliations:** Department of Pediatrics, Changyi People’s Hospital, Changyi 261300, China

**Keywords:** Congenital heart diseases (CHD), Gene polymorphisms, Methylenetetrahydrofolate reductase (MTHFR), Methionine synthase reductase (MTRR), Meta-analysis

## Abstract

**Background:** We performed the present study to better elucidate the correlations of methylenetetrahydrofolate reductase (*MTHFR*) and methionine synthase reductase (*MTRR*) gene polymorphisms with the risk of congenital heart diseases (CHD).

**Methods:** Eligible articles were searched in PubMed, Medline, Embase and CNKI. Odds ratios (ORs) and 95% confidence intervals (CIs) were used to detect any potential associations of *MTHFR* and *MTRR* gene polymorphisms with CHD.

**Results:** A total of 47 eligible studies were finally included in our meta-analysis. Our overall analyses suggested that *MTRR* rs1801394, *MTRR* rs1532268, *MTHFR* rs1801131 and *MTHFR* rs1801133 polymorphisms were all significantly associated with the risk of CHD in certain genetic models. Further subgroup analyses according to ethnicity of study participants demonstrated that the *MTRR* rs1801394 polymorphism was significantly correlated with the risk of CHD only in Asians, whereas *MTRR* rs1532268, *MTHFR* rs1801133 and *MTHFR* rs1801131 polymorphisms were significantly correlated with the risk of CHD in both Asians and Caucasians.

**Conclusions:** Our findings indicated that *MTRR* rs1532268, *MTHFR* rs1801131 and *MTHFR* rs1801133 polymorphisms may affect the risk of CHD in Asians and Caucasians, while the *MTRR* rs1801394 polymorphism may only affect in risk of CHD in Asians.

## Introduction

Congenital heart diseases (CHD) refer to a group of structural heart defects that are resulted from abnormal cardiac development. The incidence of CHD is estimated to be approximately 1% in newborns, and despite rapid advances in surgical treatments and interventional therapies over the past few decades, CHD is still the primary non-infectious cause of infant mortality worldwide [1]. Moreover, its associated complications such as heart failure, arrhythmia and sudden cardiac death may occur even after effective correction of cardiac abnormalities [2,3]. Until now, the exact cause of CHD is still largely unclear despite extensive investigations. Nevertheless, mounting evidence supports that genetic factors play a crucial part in its development. First, family clustering of CHD with variable phenotypes is not uncommon, and descendants of CHD patients suffer a higher risk of developing cardiac malformations compared with the general population [4,5]. Second, multiple genetic variants have been found to be associated with an increased risk of CHD [6–9]. Overall, these findings jointly indicate that genetic predisposition to CHD is vital for its occurrence and development.

Methylenetetrahydrofolate reductase (MTHFR) and methionine synthase reductase (MTRR) play central roles in the regulation of folate metabolism and homocysteine synthesis [10]. Previous studies have shown that taking folate supplements during pregnancy could significantly reduce the risk of cardiovascular congenital malformations in newborns [11,12]. Consequently, functional *MTHFR* and *MTRR* polymorphisms, which were known to affect plasma folate levels, were considered to be ideal candidate genetic biomarkers of CHD.

So far, numerous studies have been conducted to assess the roles of *MTHFR* and *MTRR* gene polymorphisms in CHD, but the results of these studies were controversial [13–16]. Therefore, we conducted the present meta-analysis to better evaluate potential associations of *MTHFR* and *MTRR* gene polymorphisms with the risk of CHD.

## Materials and methods

### Literature search and inclusion criteria

The current meta-analysis was adhered to the Preferred Reporting Items for Systematic Reviews and Meta-analyses (PRISMA) statement [17]. A systematic literature search of PubMed, Medline, Embase and China National Knowledge Infrastructure (CNKI) was performed to retrieve all relevant articles. The key words used in this literature search included: ‘5-methyltetrahydrofolate-homocysteine methyltransferase reductase’, ‘methionine synthase reductase’, ‘MTRR’, ‘MSR’, ‘methylenetetrahydrofolate reductase’, ‘MTHFR’, ‘polymorphism’, ‘variant’, ‘mutation’, ‘genotype’, ‘allele’, ‘congenital heart disease‘, ‘congenital heart defect’ and ‘congenital cardiovascular malformation’ (see Supplementary File S1). To identify other potentially relevant publications, we also reviewed the reference lists of all retrieved articles.

Eligible studies of the current meta-analysis must met all the following criteria: (1) evaluate potential associations of *MTRR* and/or *MTHFR* gene polymorphisms with the risk of CHD; (2) provide sufficient data to calculate odds ratios (ORs) and 95% confidence intervals (CIs); (3) full text in Chinese or English available. For duplicate reports, only the study with the largest sample size was included. Reviews, comments, letters and family-based association studies were excluded.

### Data extraction and quality assessment

The following information was extracted from each included study: name of the first author, year of publication, country and ethnicity of study subjects, type of CHD, genotypic frequencies of *MTRR* and/or *MTHFR* gene polymorphisms in cases and controls, and whether the distributions of investigated gene polymorphisms in the control group violated Hardy–Weinberg equilibrium (HWE).

The Newcastle–Ottawa scale (NOS), a classical assessment tool of observational studies that evaluates the quality of articles from three dimensions: selection, comparability and exposure, was adopted to assess the quality of included studies [18]. The NOS has a score range of 0 to 9, and studies with a score of more than 7 were considered to be of high quality.

Two reviewers (Aiping Xu and Weiping Wang) conducted data extraction and quality assessment independently. When necessary, the reviewers wrote to the corresponding authors for extra information or raw data. Disagreements between two reviewers were solved by discussion with the third reviewer (Xiaolei Jiang) until a consensus was reached.

### Statistical analysis

All data analyses in the present study were carried out using Review Manager Version 5.3.3 (The Cochrane Collaboration, Software Update, Oxford, United Kingdom). The probability value (*P* value) of HWE in the control group was calculated with the chi-square test. ORs and 95% CIs were used to estimate potential associations of *MTRR* and/or *MTHFR* gene polymorphisms with the risk of CHD in the dominant, recessive, additive and allele models, and a *P* value of 0.05 or less was considered as statistically significant. The *Q* test and *I*^2^ statistic were adopted to assess between-study heterogeneity. If *P* value of *Q* test was less than 0.1 or *I*^2^ was greater than 50%, random-effect models would be applied for analyses due to the existence of obvious heterogeneity. Otherwise, fixed-effect models would be employed for analyses. Subgroup analyses were subsequently performed according to ethnicity of study participants and type of disease. Sensitivity analyses were conducted to test the stability of the results. Publication bias was evaluated with funnel plots.

## Results

### Characteristics of included studies

**Figure 1:**
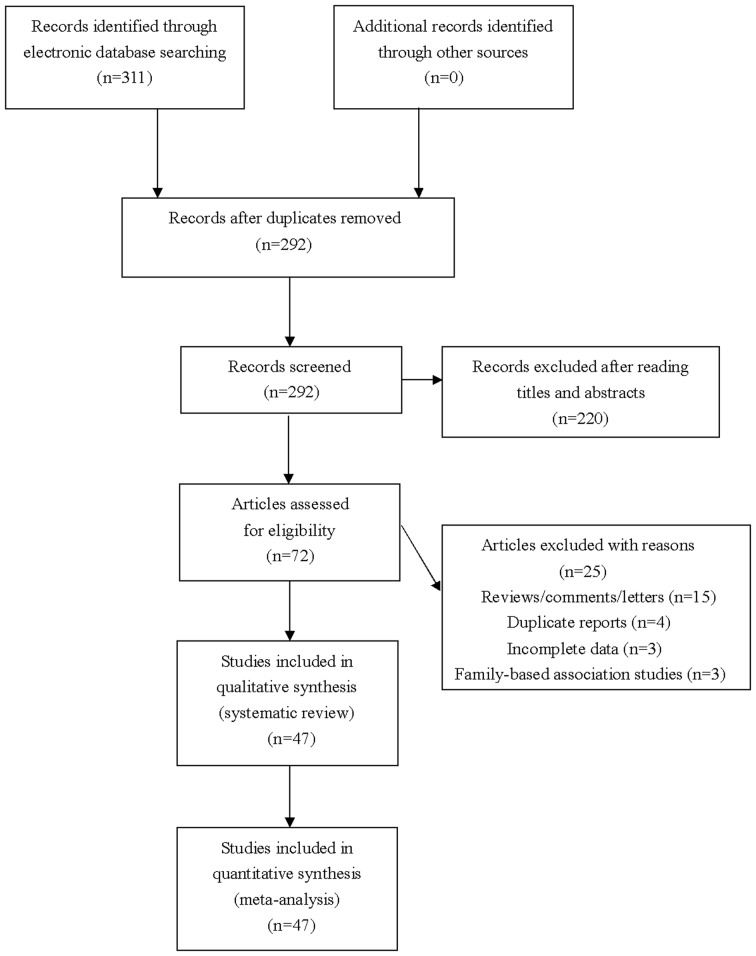
Flowchart of study selection for the present study

The literature search identified 311 citations. After exclusion of irrelevant and duplicate articles by reading titles and abstracts, 72 articles were selected for further evaluation. Another 25 articles were subsequently excluded after reading full texts, and a total of 47 studies that met the inclusion criteria were finally included in our meta-analysis (see Figure 1). Characteristics of included studies were summarized in Table 1.

**Table 1:** The characteristics of included studies

First author, year	Country	Ethnicity	Sex, male (%) Case/ Control	Age (years) Case/ Control	Type of disease	Sample size	Genotype distribution		*P*-value for HWE	NOS score
							Cases	Controls		
**rs1801394**										
Benke, 2015	Hungry	Caucasian	59.2/61.5	2.42/3.08	CHD	72/117	64/7/1	110/6/1	0.016	7
Christensen, 2013	Canada	Mixed	NA	NA	CHD	245/65	68/123/54	22/32/11	0.912	7
Gong, 2010	China	Asian	NA	NA	CHD	60/60	38/21/1	52/6/2	0.007	7
Guo, 2017	China	Asian	NA	2.31/2.48	CHD	99/114	44/46/9	67/44/3	0.174	8
Guo, 2017	China	Asian	NA	2.33/2.47	VSD	21/114	7/11/3	67/44/3	0.174	8
Hassan, 2017	Egypt	Caucasian	36.0/32.0	1.30/1.28	CHD	100/100	26/32/42	48/36/16	0.048	8
Liu, 2007	China	Asian	48.5/NA	6.50/NA	CHD	132/107	33/84/15	52/45/10	0.953	7
Locke, 2010	U.S.A.	Mixed	NA	NA	CHD	92/94	27/50/15	31/46/17	0.993	7
Noori, 2017	Iran	Caucasian	NA	NA	CHD	153/147	46/74/33	61/63/23	0.323	7
Noori, 2017	Iran	Caucasian	NA	NA	VSD	74/147	24/32/18	61/63/23	0.323	7
Noori, 2017	Iran	Caucasian	NA	NA	TOF	79/147	22/42/15	61/63/23	0.323	7
Pishva, 2013	Iran	Caucasian	46.3/44.8	4.51/5.43	VSD	123/125	41/54/28	62/53/10	0.776	7
Su, 2017	China	Asian	NA	NA	VSD	183/201	68/97/18	107/85/9	0.120	8
van Beynum, 2006	Netherlands	Caucasian	NA	NA	CHD	159/245	51/83/25	74/124/47	0.699	7
Verkleij-Hagoort, 2008	Netherlands	Caucasian	NA	1.40/1.39	CHD	229/251	79/112/38	77/122/52	0.774	7
Wang, 2013	China	Asian	NA	NA	CHD	160/188	90/59/11	105/71/12	0.999	7
Wang, 2018	China	Asian	NA	2.18/1.81	CHD	102/100	51/39/12	75/21/4	0.126	7
Weine, 2012	Russia	Caucasian	NA	2.15/2.11	CHD	51/390	19/23/9	128/191/71	0.986	7
Zeng, 2011	China	Asian	43.4/47.2	NA	CHD	599/672	309/234/56	375/253/44	0.880	8
Zhao, 2012	China	Asian	52.4/54.4	6.59/6.59	CHD	2340/2270	1308/860/172	1294/818/158	0.067	8
**rs1532268**										
Hassan, 2017	Egypt	Caucasian	36.0/32.0	1.30/1.28	CHD	100/100	14/40/46	38/36/26	0.007	8
Pishva, 2013	Iran	Caucasian	46.3/44.8	4.51/5.43	VSD	123/125	53/50/20	66/54/5	0.134	7
Su, 2017	China	Asian	NA	NA	VSD	183/201	66/96/21	105/80/16	0.889	8
Zeng, 2011	China	Asian	43.4/47.2	NA	CHD	599/672	383/201/15	476/176/20	0.450	8
**rs1801131**										
Božović, 2011	Croatia	Caucasian	49.1/49.3	1.03/2.78	CHD	54/221	30/22/2	101/98/22	0.803	7
Brandalize, 2009	Brazil	African	NA	NA	CHD	239/197	143/84/12	113/76/8	0.275	8
Chao, 2014	Taiwan	Asian	11.8/38.2	46.7/50.9	PDA	17/34	13/2/2	15/19/0	0.024	8
Christensen, 2013	Canada	Mixed	NA	NA	CHD	246/65	133/93/20	36/22/7	0.212	7
Feng, 2016	China	Asian	46.3/63.2	1.3/1.9	CHD	257/49	194/51/12	35/14/0	0.243	7
Galdieri, 2007	Brazil	African	NA	NA	CHD	57/38	35/21/1	19/16/3	0.884	7
Guo, 2017	China	Asian	NA	2.31/2.48	CHD	99/114	71/28/0	89/24/1	0.655	8
Guo, 2017	China	Asian	NA	2.33/2.48	VSD	21/114	14/7/0	89/24/1	0.655	8
Huang, 2014	China	Asian	56.1/57.5	2.54/2.70	TOF	170/206	111/56/3	146/54/6	0.712	8
Koshy, 2015	India	Caucasian	NA	NA	CHD	96/100	27/32/37	58/20/22	<0.001	7
Locke, 2010	U.S.A.	Mixed	NA	NA	CHD	87/88	42/39/6	30/49/9	0.090	7
Obermann-Borst, 2011	Netherlands	Caucasian	60.8/55.9	17.0/17.3	CHD	139/183	69/57/13	75/90/18	0.227	8
Sahiner, 2014	Turkey	Caucasian	57.1/NA	7.63/NA	CHD	137/93	45/68/24	31/54/8	0.022	8
Sayin Kocakap, 2015	Turkey	Caucasian	NA	NA	CHD	69/99	20/36/13	51/37/11	0.288	8
Shi, 2015	China	Asian	38.85/57.41	2.18/2.12	CHD	153/216	95/39/19	157/53/6	0.555	7
Storti, 2003	Italy	Caucasian	NA	2.50/2.58	CHD	100/100	43/46/11	50/43/7	0.582	7
van Driel, 2008	Netherlands	Caucasian	58.0/57.0	1.40/1.39	CHD	230/251	104/102/24	116/104/31	0.311	8
Wang, 2018	China	Asian	NA	2.18/1.81	CHD	102/100	57/40/5	60/36/4	0.624	7
Xu, 2010	China	Asian	53.7/53.0	6.50/6.69	CHD	502/527	316/168/18	326/185/16	0.091	8
Xu, 2010	China	Asian	NA	NA	VSD	257/527	169/86/2	326/185/16	0.091	8
Xu, 2010	China	Asian	NA	NA	ASD	41/527	21/16/4	326/185/16	0.091	8
Zidan, 2013	Egypt	Caucasian	NA	NA	CHD	80/80	16/27/37	30/26/24	0.002	7
**rs1801133**										
Božović, 2011	Croatia	Caucasian	49.1/49.3	1.03/2.78	CHD	54/221	20/28/6	101/97/23	0.968	7
Brandalize, 2009	Brazil	African	NA	NA	CHD	239/197	94/113/32	86/93/18	0.313	8
Chao, 2014	Taiwan	Asian	11.8/38.2	46.7/50.9	PDA	17/34	10/5/2	19/12/3	0.586	8
Christensen, 2013	Canada	Mixed	NA	NA	CHD	246/65	94/117/35	27/29/9	0.787	7
Feng, 2016	China	Asian	46.3/63.2	1.3/1.9	CHD	257/49	122/114/21	21/22/6	0.949	7
Galdieri, 2007	Brazil	African	NA	NA	CHD	58/38	30/21/7	18/14/6	0.263	7
Gong, 2012	China	Asian	65.5/61.8	1.91/1.58	CHD	244/136	45/123/76	43/72/21	0.309	9
Gong, 2012	China	Asian	61.6/61.8	1.55/1.58	TOF	120/136	21/59/40	43/72/21	0.309	9
Gong, 2012	China	Asian	69.4/61.8	2.27/1.58	TGA	124/136	24/64/36	43/72/21	0.309	9
Guo, 2017	China	Asian	NA	2.31/2.48	CHD	99/114	20/41/38	36/48/30	0.097	8
Guo, 2017	China	Asian	NA	2.33/2.48	VSD	21/114	8/8/5	36/48/30	0.097	8
Huang, 2014	China	Asian	56.1/57.5	2.54/2.70	TOF	168/204	63/45/60	84/72/48	<0.001	8
Jiang, 2015	China	Asian	NA	2.34/2.35	CHD	100/100	38/46/16	41/48/11	0.523	7
Jing, 2013	China	Asian	NA	NA	CHD	104/208	16/42/46	55/114/39	0.139	7
Junker, 2001	Germany	Caucasian	53.0/NA	16.0/NA	CHD	114/228	51/42/21	129/78/21	0.075	7
Koshy, 2015	India	Caucasian	63.5/49.0	6.51/7.61	CHD	96/90	95/1/0	83/7/0	0.701	7
Kuehl, 2010	U.S.A.	Mixed	50.4/56.0	NA	CHD	55/300	12/33/10	134/134/32	0.861	8
Lee, 2005	Taiwan	Asian	NA	NA	CHD	213/195	110/89/14	114/68/13	0.513	7
Li, 2005	China	Asian	48.4/57.2	NA	CHD	183/103	30/95/58	22/57/24	0.277	7
Li, 2013	China	Asian	54.2/57.1	2.68/2.79	CHD	144/168	26/52/66	49/84/35	0.928	7
Liu, 2007	China	Asian	48.5/NA	6.5/NA	CHD	132/107	30/68/34	46/48/13	0.930	7
Locke, 2010	U.S.A.	Mixed	NA	NA	CHD	91/94	38/39/14	49/37/8	0.787	7
Noori, 2017	Iran	Caucasian	NA	NA	CHD	153/147	95/51/7	100/46/1	0.078	7
Noori, 2017	Iran	Caucasian	NA	NA	VSD	74/147	24/32/18	100/46/1	0.078	7
Noori, 2017	Iran	Caucasian	NA	NA	TOF	79/147	22/42/15	100/46/1	0.078	7
Obermann-Borst, 2011	Netherlands	Caucasian	60.8/55.9	1.41/1.44	CHD	139/183	64/66/9	92/76/15	0.900	8
Sahiner, 2014	Turkey	Caucasian	57.1/NA	7.63/NA	CHD	136/93	69/53/14	47/39/7	0.779	8
Sayin Kocakap, 2015	Turkey	Caucasian	NA	NA	CHD	75/95	40/33/2	43/44/8	0.484	8
Shaw, 2005	China	Asian	NA	NA	CHD	153/434	69/68/16	202/180/52	0.227	7
Shi, 2015	China	Asian	38.85/57.41	2.18/2.12	CHD	153/216	55/68/30	70/101/45	0.444	7
Storti, 2003	Italy	Caucasian	NA	2.50/2.58	CHD	100/100	27/53/20	26/54/20	0.401	7
van Beynum, 2006	Netherlands	Caucasian	55.0/49.0	3.4/9.4	CHD	158/261	72/68/18	131/107/23	0.863	7
van Driel, 2008	Netherlands	Caucasian	58.0/57.0	1.40/1.39	CHD	229/251	99/103/27	119/107/25	0.895	8
Wang, 2013	China	Asian	NA	NA	CHD	160/188	59/76/25	53/100/35	0.312	7
Wang, 2016	China	Asian	NA	NA	CHD	147/168	14/73/60	49/84/35	0.928	8
Wang, 2018	China	Asian	NA	2.18/1.83	CHD	102/100	31/58/13	55/42/3	0.130	7
Xu, 2010	China	Asian	53.7/53.0	6.50/6.69	CHD	502/527	162/244/96	151/261/115	0.911	8
Xu, 2010	China	Asian	NA	NA	VSD	257/527	83/130/44	151/261/115	0.911	8
Xu, 2010	China	Asian	NA	NA	ASD	41/527	12/17/12	151/261/115	0.911	8
Xu, 2013	China	Asian	64.8/52.4	NA	CHD	228/230	73/106/49	124/74/32	<0.001	8
Yan, 2003	China	Asian	NA	NA	CHD	187/103	32/97/58	22/57/24	0.277	7
Zhou, 2012	China	Asian	48.5/57.8	NA	TOF	136/277	23/60/53	88/126/63	0.168	8
Zhu, 2006	China	Asian	35.1/57.7	6.2/8.4	CHD	56/103	7/22/27	22/57/24	0.277	7
Zhu, 2006	China	Asian	NA	NA	ASD	22/103	3/7/12	22/57/24	0.277	7
Zhu, 2006	China	Asian	NA	NA	PDA	34/103	4/15/15	22/57/24	0.277	7
Zidan, 2013	Egypt	Caucasian	NA	NA	CHD	80/80	18/21/41	32/21/27	<0.001	7

Abbreviations: ASD, atrial septal defect; CHD, congenital heart disease; HWE, Hardy–Weinberg equilibrium; NA, not available; NOS, Newcastle–Ottawa scale; PDA, patent ductus arteriosus; TGA, transposition of the great arteries; TOF, tetralogy of fallot; VSD, ventricular septal defect.

### Overall and subgroup analyses for *MTRR* polymorphisms

To investigate potential associations between *MTRR* gene polymorphisms and the risk of CHD, 17 studies about rs1801394 polymorphism and 4 studies about rs1532268 polymorphism were enrolled for overall analyses. Significant associations with the risk of CHD were detected for rs1801394 (dominant model: *P*=0.0001, OR = 0.68, 95%CI 0.56–0.83; recessive model: *P*=0.009, OR = 1.40, 95%CI 1.09–1.79; additive model: *P*=0.008, OR = 1.12, 95%CI 1.03-1.21; allele model: *P*=0.0001, OR = 0.73, 95%CI 0.63–0.86) and rs1532268 (dominant model: *P*=0.001, OR = 0.56, 95%CI 0.39–0.80; additive model: *P*=0.0009, OR = 1.36, 95%CI 1.13–1.63; allele model: *P*=0.0006, OR = 0.61, 95%CI 0.47–0.81) polymorphisms in overall analyses. Further subgroup analyses according to ethnicity of study participants demonstrated that the rs1801394 polymorphism was significantly correlated with the risk of CHD only in Asians, whereas the rs1532268 polymorphism was significantly correlated with the risk of CHD in both Asians and Caucasians. When we stratified data based on type of disease, we found that both rs1801394 and rs1532268 polymorphisms were significantly associated with the risk of VSD (see Table 2 and Supplementary Figure S1).

**Table 2:** Results of overall and subgroup analyses

Population	Sample size	Dominant comparison			Recessive comparison			Additive comparison			Allele comparison		
		*P* value	OR (95%CI)	*I*^2^ statistic	*P* value	OR (95%CI)	*I*^2^ statistic	*P* value	OR (95%CI)	*I*^2^ statistic	*P* value	OR (95%CI)	*I*^2^ statistic
**rs1801394**													
Overall	4899/5246	**0.0001**	**0.68 (0.56–0.83)**	72%	**0.009**	**1.40 (1.09–1.79)**	56%	**0.008**	**1.12 (1.03–1.21)**	48%	**0.0001**	**0.73 (0.63–0.86)**	77%
Caucasian	887/1375	0.11	0.75 (0.52–1.07)	68%	0.18	1.45 (0.85**–**2.49)	76%	0.62	1.05 (0.87**–**1.26)	0%	0.10	0.75 (0.53**–**1.06)	84%
Asian	3675/3712	**0.0008**	**0.60 (0.45–0.81)**	82%	**0.02**	**1.24 (1.04–1.48)**	34%	**0.006**	**1.43 (1.11–1.85)**	74%	**0.0007**	**0.69 (0.55–0.85)**	79%
VSD	558/587	**<0.0001**	**0.55 (0.43–0.69)**	0%	**<0.0001**	**2.22 (1.51–3.26)**	15%	**0.02**	**1.32 (1.04–1.66)**	0%	**<0.0001**	**0.60 (0.51–0.72)**	0%
**rs1532268**													
Overall	1005/1098	**0.001**	**0.56 (0.39–0.80)**	64%	0.06	1.83 (0.96**–**3.48)	68%	**0.0009**	**1.36 (1.13–1.63)**	23%	**0.0006**	**0.61 (0.47–0.81)**	68%
Caucasian	223/225	0.08	0.44 (0.18–1.09)	78%	**<0.0001**	**2.93 (1.77–4.88)**	16%	0.94	1.02 (0.70**–**1.48)	0%	**<0.0001**	**0.50 (0.38–0.66)**	46%
Asian	782/873	**0.008**	**0.64 (0.46–0.89)**	52%	0.65	1.12 (0.69**–**1.80)	29%	**0.0002**	**1.48 (1.21–1.83)**	0%	**0.0007**	**0.75 (0.63–0.88)**	33%
VSD	306/326	**<0.0001**	**0.58 (0.42–0.79)**	0%	0.11	2.48 (0.82**–**7.52)	70%	0.47	1.25 (0.68**–**2.28)	71%	**<0.0001**	**0.62 (0.49–0.79)**	0%
**rs1801131**													
Overall	2834/2761	0.44	0.93 (0.76–1.13)	63%	**0.003**	**1.36 (1.11–1.67)**	**42%**	0.88	0.99 (0.88**–**1.11)	38%	0.23	0.90 (0.75**–**1.07)	72%
Caucasian	905/1127	0.14	0.74 (0.49–1.10)	78%	**0.01**	**1.40 (1.08–1.81)**	**45%**	0.62	1.05 (0.87**–**1.26)	43%	0.10	0.77 (0.57**–**1.05)	81%
Asian	1300/1246	0.75	0.97 (0.82–1.15)	23%	**0.009**	**1.78 (1.15–2.75)**	**47%**	0.97	0.99 (0.74**–**1.34)	55%	0.42	0.89 (0.68**–**1.18)	64%
VSD	601/641	0.95	0.99 (0.79–1.25)	21%	0.74	1.12 (0.58**–**2.18)	0%	0.96	0.99 (0.78**–**1.26)	44%	0.88	0.98 (0.81**–**1.20)	0%
**rs1801133**													
Overall	5508/6207	**<0.0001**	**0.73 (0.63–0.84)**	62%	**<0.0001**	**1.54 (1.30–1.83)**	59%	0.86	1.01 (0.93**–**1.09)	86%	**<0.0001**	**0.75 (0.67–0.84)**	73%
Caucasian	1334/1749	**0.02**	**0.83 (0.72–0.97)**	26%	**0.01**	**1.35 (1.06–1.72)**	28%	0.42	1.06 (0.91**–**1.24)	0%	**0.004**	**0.84 (0.75–0.95)**	50%
Asian	3485/3764	**<0.0001**	**0.67 (0.55–0.83)**	73%	**<0.0001**	**1.66 (1.32–2.10)**	71%	0.43	0.96 (0.87**–**1.06)	48%	**<0.0001**	**0.70 (0.60–0.83)**	81%
TOF	701/764	**0.0001**	**0.63 (0.50–0.80)**	48%	**<0.0001**	**2.17 (1.66–2.84)**	0%	0.28	0.89 (0.71**–**1.10)	0%	**<0.0001**	**0.62 (0.53–0.72)**	4%
VSD	754/788	0.48	0.85 (0.54–1.33)	68%	0.37	1.43 (0.65**–**3.14)	75%	0.90	0.99 (0.81**–**1.21)	0%	0.36	0.82 (0.54**–**1.25)	80%

Abbreviations: ASD, atrial septal defect; CHD, congenital heart disease; CI, confidence interval; NA, not available; OR, odds ratio; PDA, patent ductus arteriosus; TOF, tetralogy of fallot; VSD, ventricular septal defect.

The values in bold represent there is statistically significant differences between cases and controls.

### Overall and subgroup analyses for *MTHFR* polymorphisms

To investigate potential associations between *MTHFR* gene polymorphisms and the risk of CHD, 19 studies about rs1801131 polymorphism and 37 studies about rs1801133 polymorphism were enrolled for overall analyses. Significant associations with the risk of CHD were detected for rs1801131 (recessive model: *P*=0.003, OR = 1.36, 95%CI 1.11–1.67) and rs1801133 (dominant model: *P*<0.0001, OR = 0.73, 95%CI 0.63–0.84; additive model: *P*<0.0001, OR = 1.54, 95%CI 1.30–1.83; allele model: *P*<0.0001, OR = 0.75, 95%CI 0.67–0.84) polymorphisms in overall analyses. Further subgroup analyses according to ethnicity of study participants demonstrated that rs1801133 and rs1801131 polymorphisms were significantly correlated with the risk of CHD in both Asians and Caucasians. When we stratified data based on type of disease, we found that the rs1801133 polymorphism was significantly associated with the risk of TOF (see Table 2 and Supplementary Figure S1).

### Sensitivity analyses

To examine stabilities of synthetic results, sensitivity analyses were further performed by removing studies that departed from HWE. No changes of results were detected for investigated gene polymorphisms in any comparisons, which indicated that our findings were quite statistically stable.

### Publication biases

Funnel plots were used to assess potential publication biases in the present study. No apparent asymmetry of funnel plots was observed in any comparisons, which suggested that our findings were unlikely to be influenced by obvious publication biases (see Supplementary Figure S2).

## Discussion

CHD contain various structural cardiovascular malformations that are actually or potentially of functional significances [19]. Historically, few CHD patients reached adulthood, but thanks to enormous advances in interventional therapies and surgical treatments over the past few years, the average life expectancy of CHD patients has been significantly improved [20]. However, despite substantially improved prognosis, CHD remains to be the leading cause of infant deaths all over the world.

MTHFR and MTRR are fundamental regulatory enzymes of folate and homocysteine metabolism. Considering the consistently observed association between folic acid consumption and a reduced risk of cardiac deformity, functional polymorphisms of *MTHFR* and *MTRR*, which were known to be associated with altered enzymatic activities, were thought to be correlated with the risk of CHD [11,12]. Recently, several studies have tried to explore the potential associations of functional *MTHFR* and *MTRR* gene polymorphisms with the risk of CHD, but the results of these studies were inconsistent. Therefore, we conducted the present meta-analysis to obtain a more conclusive result. Our overall analyses suggested that *MTRR* rs1801394, *MTRR* rs1532268, *MTHFR* rs1801131 and *MTHFR* rs1801133 polymorphisms were all significantly associated with the risk of CHD in certain genetic models. Further subgroup analyses according to ethnicity of study participants demonstrated that the *MTRR* rs1801394 polymorphism was significantly correlated with the risk of CHD only in Asians, whereas *MTRR* rs1532268, *MTHFR* rs1801133 and *MTHFR* rs1801131 polymorphisms were significantly correlated with the risk of CHD in both Asians and Caucasians. When we stratified data based on type of disease, we found that both *MTRR* rs1801394 and *MTRR* rs1532268 polymorphisms were significantly associated with the risk of VSD, whereas the *MTHFR* rs1801133 polymorphism was significantly associated with the risk of TOF. The stabilities of synthetic results were subsequently evaluated in sensitivity analyses, and no changes of results were observed in any comparisons, which indicated that our findings were quite stable and reliable. It is noteworthy that obvious between-study heterogeneities were detected in several comparisons. However, a great reduction in heterogeneities was found in further stratified analyses, which suggested that differences in ethnic background and type of disease could partially explain the observed heterogeneities.

Our meta-analysis is certainly not without limitations. First, our results were based on unadjusted estimations, and lack of analyses adjusted for potential confounding factors such as age, sex and co-morbidity conditions may impact the reliability of our findings. Second, heterogeneity remained significant in certain subgroups, which suggested that the conflicting results of eligible studies could not be fully explained by differences in ethnicity of study population or type of CHD, and other unmeasured characteristics of study participants may also attribute to the observed between-study heterogeneities. Third, associations between investigated polymorphisms and the risk of CHD may also be influenced by gene–gene and gene–environmental interactions. However, we failed to analyze the effect of these interactions in our study because only very little relevant data were provided by enrolled literatures. Taken these limitations into consideration, the results obtained by the present study should be interpreted with caution.

In conclusion, the current meta-analysis indicated that *MTRR* rs1801394, *MTRR* rs1532268, *MTHFR* rs1801131 and *MTHFR* rs1801133 polymorphisms may affect the risk of CHD in Asians and Caucasians, while the *MTRR* rs1801394 polymorphism may only affect in risk of CHD in Asians. However, it is notable that relevant studies were still at the early stage and further well-designed studies are still warranted to confirm our findings.

## Supporting information

**Figure:** Funnel plots of investigated polymorphisms

**Table:** Funnel plots of investigated polymorphisms

## Abbreviations

CHD: congenital heart diseases
CI: confidence interval
HWE: Hardy–Weinberg equilibrium
MTHFR: methylenetetrahydrofolate reductase
MTRR: methionine synthase reductase
NOS: Newcastle–Ottawa scale
OR: odds ratio
