# Supplementary material for: The roles of MTRR and MTHFR gene polymorphisms in congenital heart diseases: a meta-analysis
Source: Biosci Rep. 2018 Dec 7;38(6):BSR20181160. doi: 10.1042/BSR20181160 (PMC6435561; doi:10.1042/BSR20181160)
Supplement: Supplementary file 1 [file bsr20181160_Supp1.pdf]

For relationship between *MTHFR* polymorphisms and CHD:

(methylenetetrahydrofolate reductase OR MTHFR) AND (polymorphism OR variant OR mutation OR genotype OR allele) AND (congenital heart disease OR congenital heart defect OR congenital cardiovascular malformation)

For relationship between *MTRR* polymorphisms and CHD:

(5-methyltetrahydrofolate-homocysteine methyltransferase reductase OR methionine synthase reductase OR MTRR OR MSR) AND (polymorphism OR variant OR mutation OR genotype OR allele) AND (congenital heart disease OR congenital heart defect OR congenital cardiovascular malformation)

Forest plots of investigated polymorphisms

Study or Subgroup	Experimental Events	Experimental Total	Control Events	Control Total	Weight	Odds Ratio M-H, Random, 95% CI
Benke 2015	64	72	110	117	2.5%	0.51 [0.18, 1.47]
Christensen 2013	68	245	22	65	5.2%	0.75 [0.42, 1.35]
Gong 2010	38	60	52	60	3.1%	0.27 [0.11, 0.66]
Guo 2017	44	99	67	114	5.6%	0.56 [0.33, 0.97]
Hassan 2017	26	100	48	100	5.1%	0.38 [0.21, 0.69]
Liu 2007	33	132	52	107	5.5%	0.35 [0.20, 0.61]
Locke 2010	27	92	31	94	4.9%	0.84 [0.45, 1.57]
Noori 2017	46	153	61	147	6.2%	0.61 [0.38, 0.98]
Pishva 2013	41	123	62	125	5.9%	0.51 [0.30, 0.85]
Su 2017	68	183	107	201	6.9%	0.52 [0.35, 0.78]
van Beynum 2006	51	159	74	245	6.7%	1.09 [0.71, 1.68]
Verkleij-Hagoort 2008	79	229	77	251	7.2%	1.19 [0.81, 1.74]
Wang 2013	90	160	105	188	6.7%	1.02 [0.66, 1.55]
Wang 2018	51	102	75	100	5.1%	0.33 [0.18, 0.61]
Weine 2012	19	51	128	390	5.0%	1.22 [0.66, 2.23]
Zeng 2011	309	599	375	672	8.8%	0.84 [0.68, 1.05]
Zhao 2012	1308	2340	1294	2270	9.6%	0.96 [0.85, 1.07]
**Total (95% CI)**		**4899**		**5246**	**100.0%**	**0.68 [0.56, 0.83]**
Total events	2362		2740			

Heterogeneity: Tau² = 0.10; Chi² = 57.23, df = 16 (P < 0.00001); I² = 72%
Test for overall effect: Z = 3.86 (P = 0.0001)

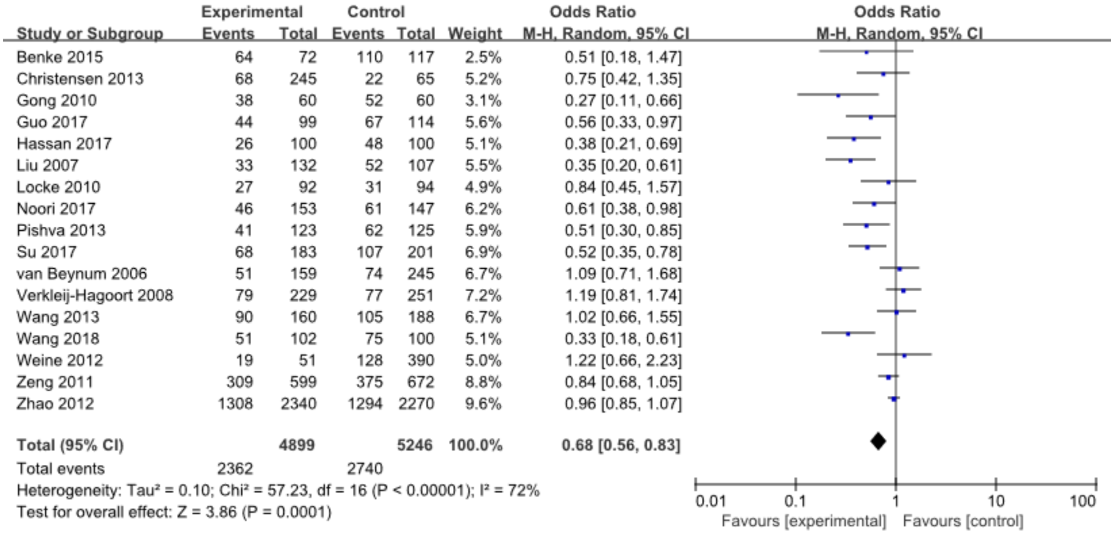

Forest plot of **rs1801394** polymorphism and CHD under dominant comparison

Study or Subgroup	Experimental Events	Experimental Total	Control Events	Control Total	Weight	Odds Ratio M-H, Random, 95% CI
Benke 2015	1	72	1	117	0.8%	1.63 [0.10, 26.53]
Christensen 2013	54	245	11	65	6.3%	1.39 [0.68, 2.84]
Gong 2010	1	60	2	60	1.0%	0.49 [0.04, 5.57]
Guo 2017	9	99	3	114	2.8%	3.70 [0.97, 14.07]
Hassan 2017	42	100	16	100	6.7%	3.80 [1.95, 7.40]
Liu 2007	15	132	10	107	5.2%	1.24 [0.53, 2.89]
Locke 2010	15	92	17	94	5.9%	0.88 [0.41, 1.89]
Noori 2017	33	153	23	147	7.5%	1.48 [0.82, 2.67]
Pishva 2013	28	123	10	125	5.8%	3.39 [1.57, 7.33]
Su 2017	18	183	9	201	5.4%	2.33 [1.02, 5.32]
van Beynum 2006	25	159	47	245	8.1%	0.79 [0.46, 1.34]
Verkleij-Hagoort 2008	38	229	52	251	8.9%	0.76 [0.48, 1.21]
Wang 2013	11	160	12	188	5.2%	1.08 [0.46, 2.53]
Wang 2018	12	102	4	100	3.4%	3.20 [1.00, 10.29]
Weine 2012	9	51	71	390	5.9%	0.96 [0.45, 2.07]
Zeng 2011	56	599	44	672	9.6%	1.47 [0.98, 2.22]
Zhao 2012	172	2340	158	2270	11.7%	1.06 [0.85, 1.33]
**Total (95% CI)**		**4899**		**5246**	**100.0%**	**1.40 [1.09, 1.79]**
Total events	539		490			

Heterogeneity: Tau² = 0.13; Chi² = 36.71, df = 16 (P = 0.002); I² = 56%
Test for overall effect: Z = 2.62 (P = 0.009)

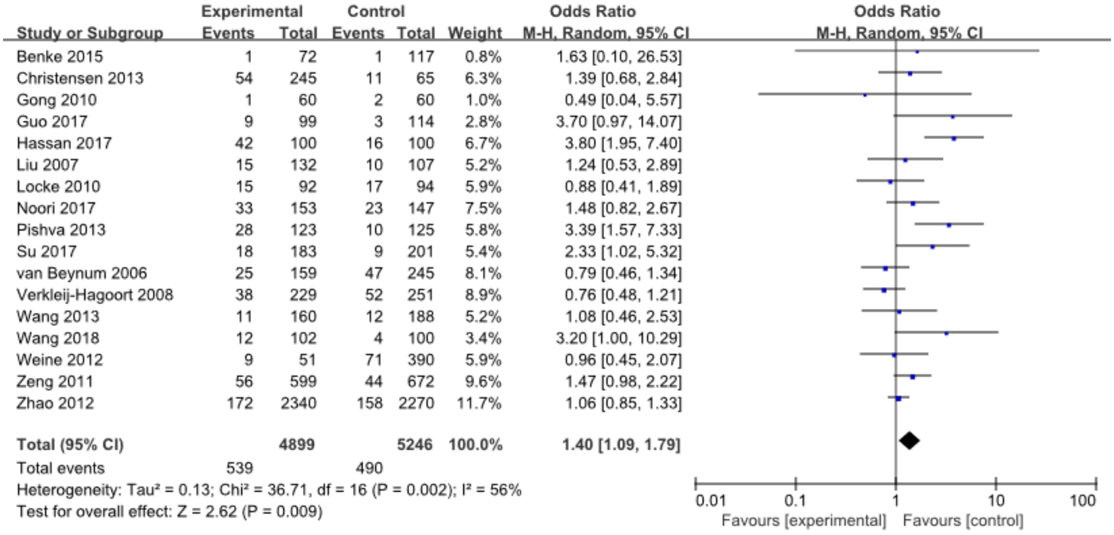

Forest plot of **rs1801394** polymorphism and CHD under recessive comparison

Study or Subgroup	Experimental Events	Experimental Total	Control Events	Control Total	Weight	Odds Ratio M-H, Fixed, 95% CI
Benke 2015	7	72	6	117	0.4%	1.99 [0.64, 6.18]
Christensen 2013	123	245	32	65	2.3%	1.04 [0.60, 1.80]
Gong 2010	21	60	6	60	0.4%	4.85 [1.79, 13.13]
Guo 2017	46	99	44	114	2.0%	1.38 [0.80, 2.38]
Hassan 2017	32	100	36	100	2.3%	0.84 [0.47, 1.50]
Liu 2007	84	132	45	107	1.7%	2.41 [1.43, 4.07]
Locke 2010	50	92	46	94	1.9%	1.24 [0.70, 2.21]
Noori 2017	74	153	63	147	3.1%	1.25 [0.79, 1.97]
Pishva 2013	54	123	53	125	2.7%	1.06 [0.64, 1.76]
Su 2017	97	183	85	201	3.5%	1.54 [1.03, 2.30]
van Beynum 2006	83	159	124	245	4.3%	1.07 [0.71, 1.59]
Verkleij-Hagoort 2008	112	229	122	251	5.5%	1.01 [0.71, 1.45]
Wang 2013	59	160	71	188	3.8%	0.96 [0.62, 1.49]
Wang 2018	39	102	21	100	1.2%	2.33 [1.25, 4.35]
Weine 2012	23	51	191	390	2.3%	0.86 [0.48, 1.54]
Zeng 2011	234	599	253	672	13.5%	1.06 [0.85, 1.33]
Zhao 2012	860	2340	818	2270	48.9%	1.03 [0.91, 1.16]
**Total (95% CI)**		**4899**		**5246**	**100.0%**	**1.12 [1.03, 1.21]**
Total events	1998		2016			

Heterogeneity: $Chi^2 = 30.85$, df = 16 (P = 0.01); $I^2 = 48\%$
Test for overall effect: Z = 2.67 (P = 0.008)

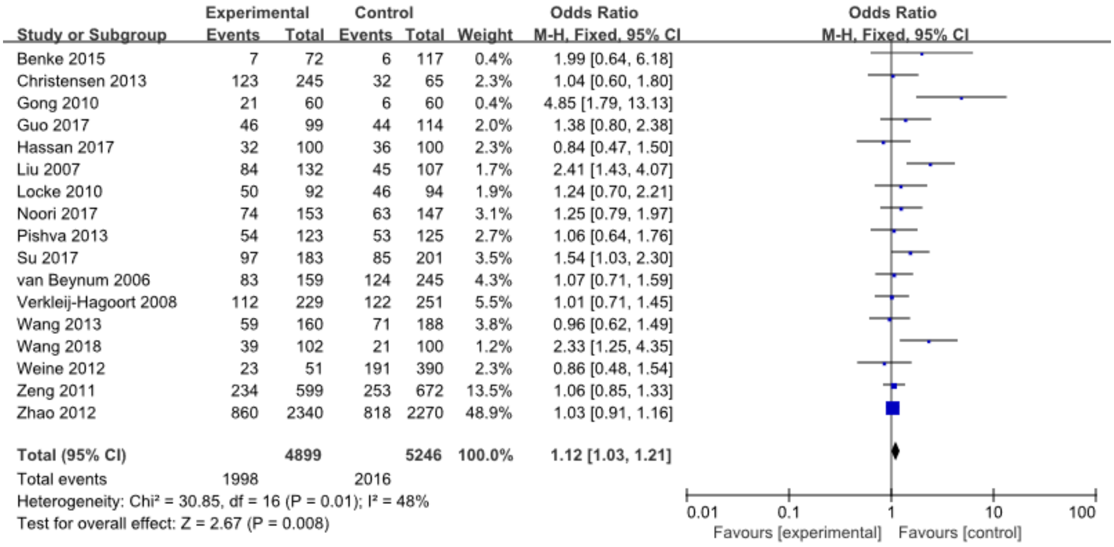

Forest plot of **rs1801394** polymorphism and CHD under additive comparison

Study or Subgroup	Experimental Events	Experimental Total	Control Events	Control Total	Weight	Odds Ratio M-H, Random, 95% CI
Benke 2015	135	144	226	234	2.0%	0.53 [0.20, 1.41]
Christensen 2013	259	490	76	130	5.8%	0.80 [0.54, 1.18]
Gong 2010	97	120	110	120	2.8%	0.38 [0.17, 0.85]
Guo 2017	134	198	178	228	5.4%	0.59 [0.38, 0.91]
Hassan 2017	84	200	132	200	5.7%	0.37 [0.25, 0.56]
Liu 2007	150	264	149	214	5.9%	0.57 [0.39, 0.84]
Locke 2010	104	184	108	188	5.6%	0.96 [0.64, 1.45]
Noori 2017	166	306	185	294	6.5%	0.70 [0.50, 0.97]
Pishva 2013	136	246	177	250	6.0%	0.51 [0.35, 0.74]
Su 2017	233	366	299	402	6.7%	0.60 [0.44, 0.82]
van Beynum 2006	185	318	272	490	7.0%	1.11 [0.84, 1.48]
Verkleij-Hagoort 2008	270	458	276	502	7.3%	1.18 [0.91, 1.52]
Wang 2013	239	320	281	376	6.3%	1.00 [0.71, 1.41]
Wang 2018	141	204	171	200	4.8%	0.38 [0.23, 0.62]
Weine 2012	61	102	447	780	5.5%	1.11 [0.73, 1.69]
Zeng 2011	852	1198	1003	1344	8.1%	0.84 [0.70, 1.00]
Zhao 2012	3476	4680	3406	4540	8.7%	0.96 [0.88, 1.06]
**Total (95% CI)**		**9798**		**10492**	**100.0%**	**0.73 [0.63, 0.86]**
Total events	6722		7496			

Heterogeneity: $Tau^2 = 0.07$; $Chi^2 = 70.27$, df = 16 (P < 0.00001); $I^2 = 77\%$
Test for overall effect: Z = 3.82 (P = 0.0001)

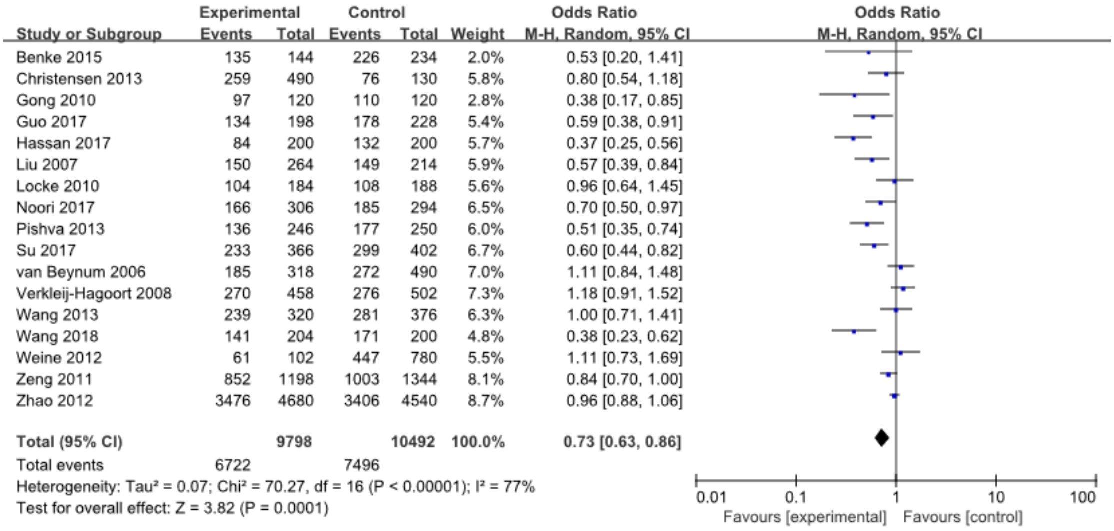

Forest plot of **rs1801394** polymorphism and CHD under allele comparison

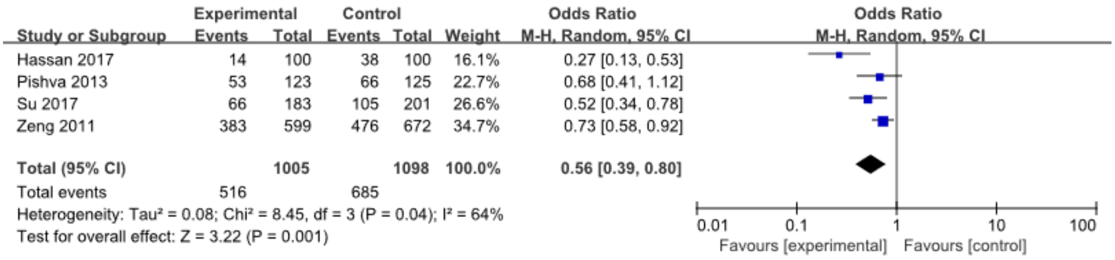

Forest plot of **rs1532268** polymorphism and CHD under dominant comparison

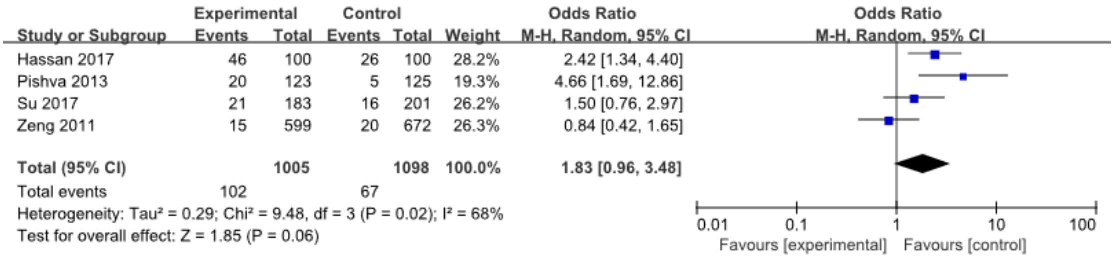

Forest plot of **rs1532268** polymorphism and CHD under recessive comparison

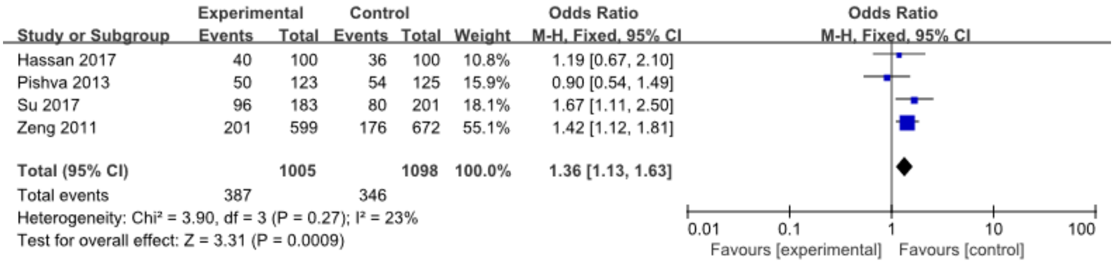

Forest plot of **rs1532268** polymorphism and CHD under additive comparison

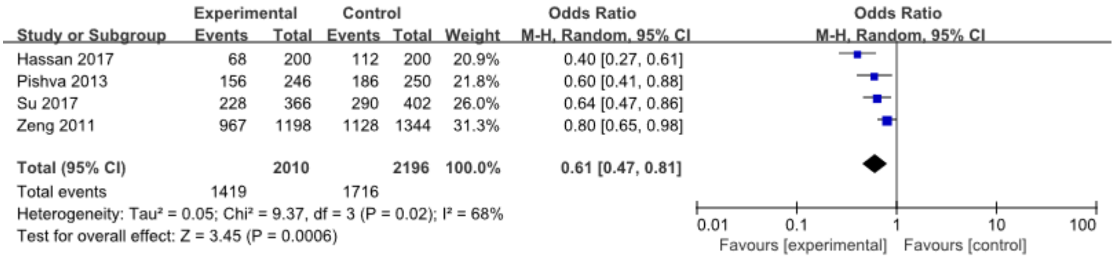

Forest plot of **rs1532268** polymorphism and CHD under allele comparison

Study or Subgroup	Experimental Events	Experimental Total	Control Events	Control Total	Weight	Odds Ratio M-H, Random, 95% CI
Božović 2011	30	54	101	221	5.0%	1.49 [0.82, 2.70]
Brandalize 2009	143	239	113	197	6.8%	1.11 [0.76, 1.62]
Chao 2014	13	17	15	34	1.8%	4.12 [1.11, 15.25]
Christensen 2013	133	246	36	65	5.4%	0.95 [0.55, 1.64]
Feng 2016	194	257	35	49	4.4%	1.23 [0.62, 2.44]
Galdieri 2007	35	57	19	38	3.5%	1.59 [0.69, 3.65]
Guo 2017	71	99	89	114	4.8%	0.71 [0.38, 1.33]
Huang 2014	111	170	146	206	6.3%	0.77 [0.50, 1.20]
Koshy 2015	27	96	58	100	5.0%	0.28 [0.16, 0.51]
Locke 2010	42	87	30	88	4.9%	1.80 [0.98, 3.32]
Obermann-Borst 2011	69	139	75	183	6.3%	1.42 [0.91, 2.21]
Sahiner 2014	45	137	31	93	5.3%	0.98 [0.56, 1.71]
Sayin Kocakap 2015	20	69	51	99	4.6%	0.38 [0.20, 0.74]
Shi 2015	95	153	137	216	6.4%	0.94 [0.62, 1.45]
Storti 2003	43	100	50	100	5.3%	0.75 [0.43, 1.32]
van Driel 2008	104	230	116	251	7.0%	0.96 [0.67, 1.38]
Wang 2018	57	102	60	100	5.3%	0.84 [0.48, 1.48]
Xu 2010	316	502	326	527	8.0%	1.05 [0.81, 1.35]
Zidan 2013	16	80	30	80	4.2%	0.42 [0.20, 0.85]
**Total (95% CI)**		**2834**		**2761**	**100.0%**	**0.93 [0.76, 1.13]**
Total events	1564		1518			

Heterogeneity: Tau² = 0.11; Chi² = 48.48, df = 18 (P = 0.0001); I² = 63%
Test for overall effect: Z = 0.77 (P = 0.44)

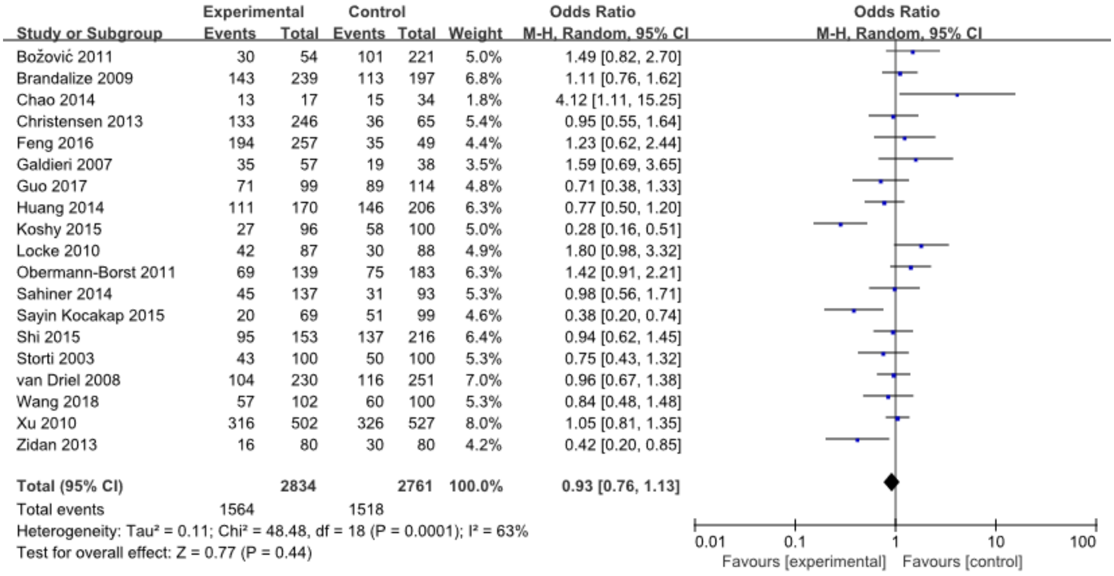

Forest plot of **rs1801131** polymorphism and CHD under dominant comparison

Study or Subgroup	Experimental Events	Experimental Total	Control Events	Control Total	Weight	Odds Ratio M-H, Fixed, 95% CI
Božović 2011	2	54	22	221	5.3%	0.35 [0.08, 1.53]
Brandalize 2009	12	239	8	197	5.3%	1.25 [0.50, 3.12]
Chao 2014	2	17	0	34	0.2%	11.13 [0.50, 245.80]
Christensen 2013	20	246	7	65	6.4%	0.73 [0.30, 1.82]
Feng 2016	12	257	0	49	0.5%	5.04 [0.29, 86.55]
Galdieri 2007	1	57	3	38	2.2%	0.21 [0.02, 2.08]
Guo 2017	0	99	1	114	0.9%	0.38 [0.02, 9.44]
Huang 2014	3	170	6	206	3.4%	0.60 [0.15, 2.43]
Koshy 2015	37	96	22	100	8.4%	2.22 [1.19, 4.16]
Locke 2010	6	87	9	88	5.3%	0.65 [0.22, 1.91]
Obermann-Borst 2011	13	139	18	183	8.9%	0.95 [0.45, 2.00]
Sahiner 2014	24	137	8	93	5.0%	2.26 [0.97, 5.27]
Sayin Kocakap 2015	13	69	11	99	4.6%	1.86 [0.78, 4.43]
Shi 2015	19	153	6	216	2.8%	4.96 [1.93, 12.74]
Storti 2003	11	100	7	100	3.9%	1.64 [0.61, 4.42]
van Driel 2008	24	230	31	251	16.8%	0.83 [0.47, 1.46]
Wang 2018	5	102	4	100	2.4%	1.24 [0.32, 4.75]
Xu 2010	18	502	16	527	9.5%	1.19 [0.60, 2.36]
Zidan 2013	37	80	24	80	8.2%	2.01 [1.05, 3.84]
**Total (95% CI)**		**2834**		**2761**	**100.0%**	**1.36 [1.11, 1.67]**
Total events	259		203			

Heterogeneity: Chi² = 30.96, df = 18 (P = 0.03); I² = 42%
Test for overall effect: Z = 2.92 (P = 0.003)

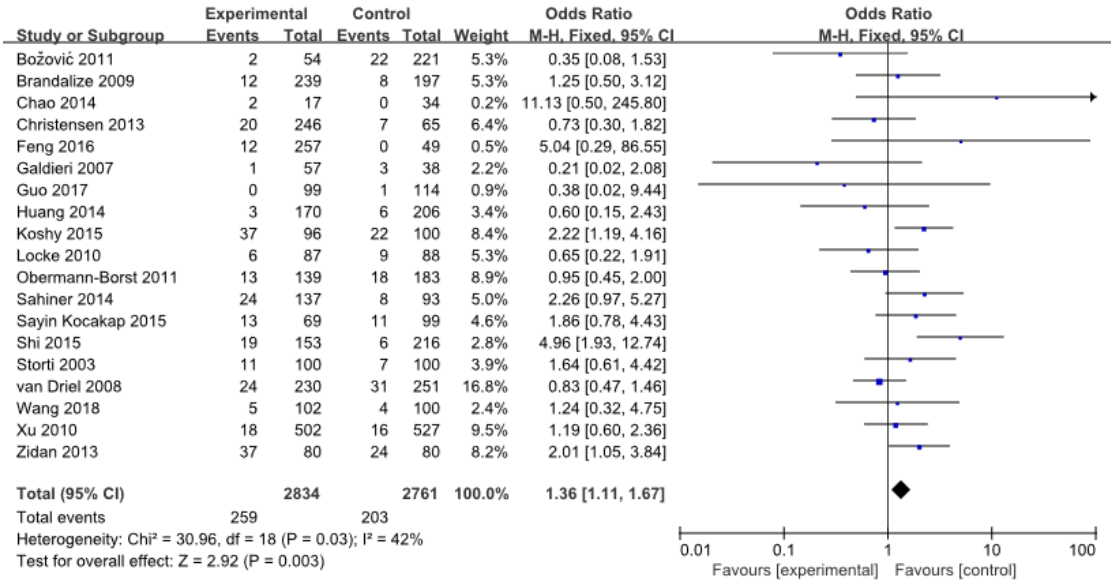

Forest plot of **rs1801131** polymorphism and CHD under recessive comparison

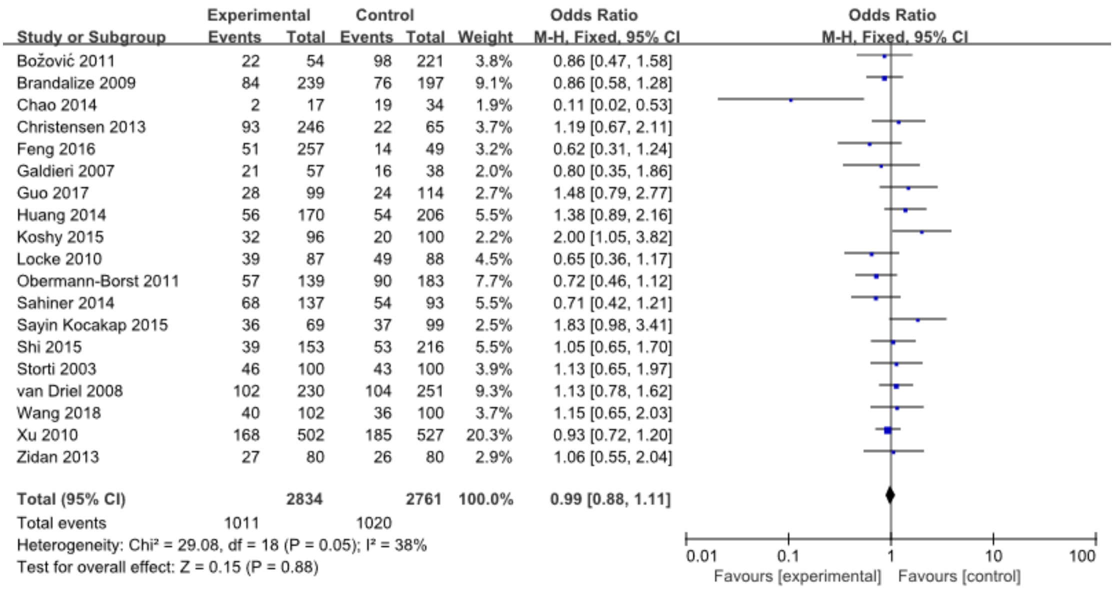

Forest plot of **rs1801131** polymorphism and CHD under additive comparison

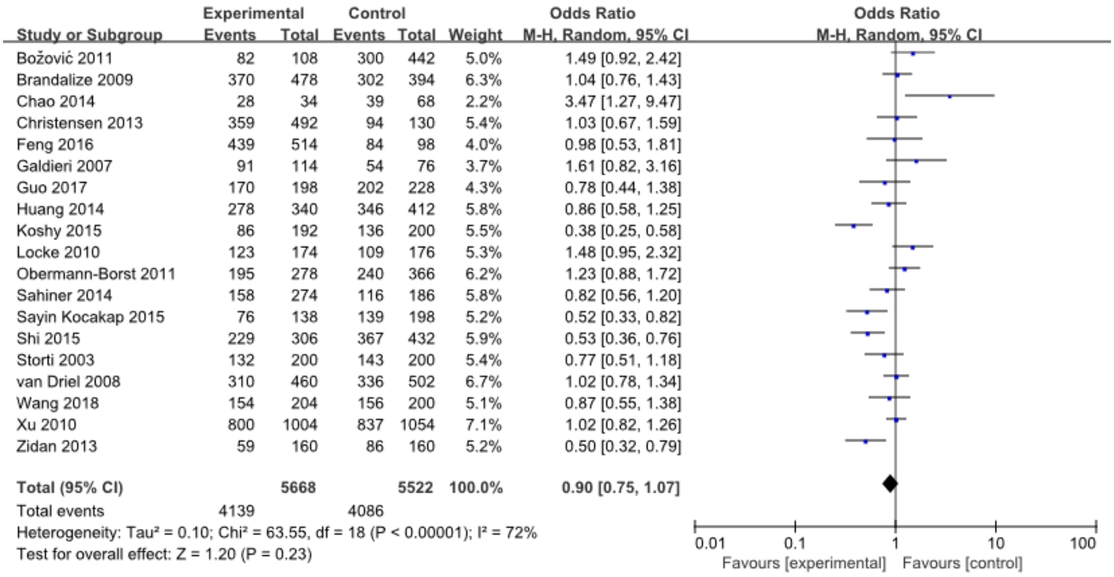

Forest plot of **rs1801131** polymorphism and CHD under allele comparison

Study or Subgroup	Experimental Events	Experimental Total	Control Events	Control Total	Weight	Odds Ratio M-H, Fixed, 95% CI
Božović 2011	20	54	101	221	1.9%	0.70 [0.38, 1.29]
Brandalize 2009	94	239	86	197	4.2%	0.84 [0.57, 1.23]
Chao 2014	10	17	19	34	0.4%	1.13 [0.35, 3.67]
Christensen 2013	94	246	27	65	2.0%	0.87 [0.50, 1.52]
Feng 2016	122	257	21	49	1.4%	1.20 [0.65, 2.23]
Galdieri 2007	30	58	18	38	0.8%	1.19 [0.52, 2.70]
Gong 2012	45	244	43	136	3.3%	0.49 [0.30, 0.79]
Guo 2017	20	99	36	114	2.0%	0.55 [0.29, 1.03]
Huang 2014	63	168	84	204	3.5%	0.86 [0.56, 1.30]
Jiang 2015	38	100	41	100	1.9%	0.88 [0.50, 1.56]
Jing 2013	16	104	55	208	2.3%	0.51 [0.27, 0.94]
Junker 2001	51	114	129	228	3.5%	0.62 [0.40, 0.98]
Koshy 2015	95	96	83	90	0.1%	8.01 [0.97, 66.48]
Kuehl 2010	12	55	134	300	2.4%	0.35 [0.18, 0.68]
Lee 2005	110	213	114	195	4.3%	0.76 [0.51, 1.12]
Li 2005	30	183	22	103	1.7%	0.72 [0.39, 1.33]
Li 2013	26	144	49	168	2.7%	0.54 [0.31, 0.92]
Liu 2007	30	132	46	107	2.9%	0.39 [0.22, 0.68]
Locke 2010	38	91	49	94	2.1%	0.66 [0.37, 1.18]
Noori 2017	95	153	100	147	2.9%	0.77 [0.48, 1.24]
Obermann-Borst 2011	64	139	92	183	3.2%	0.84 [0.54, 1.31]
Sahiner 2014	69	136	47	93	2.0%	1.01 [0.59, 1.71]
Sayin Kocakap 2015	40	75	43	95	1.3%	1.38 [0.75, 2.54]
Shaw 2005	69	153	202	434	4.3%	0.94 [0.65, 1.37]
Shi 2015	55	153	70	216	2.8%	1.17 [0.76, 1.81]
Storti 2003	27	100	26	100	1.4%	1.05 [0.56, 1.97]
van Beynum 2006	72	158	131	261	4.0%	0.83 [0.56, 1.23]
van Driel 2008	99	229	119	251	4.8%	0.84 [0.59, 1.21]
Wang 2013	59	160	53	188	2.3%	1.49 [0.95, 2.34]
Wang 2016	14	147	49	168	3.1%	0.26 [0.13, 0.49]
Wang 2018	31	102	55	100	2.9%	0.36 [0.20, 0.64]
Xu 2010	162	502	151	527	7.4%	1.19 [0.91, 1.55]
Xu 2013	73	228	124	230	6.2%	0.40 [0.28, 0.59]
Yan 2003	32	187	22	103	1.7%	0.76 [0.41, 1.39]
Zhou 2012	23	136	88	277	3.6%	0.44 [0.26, 0.73]
Zhu 2006	7	56	22	103	1.0%	0.53 [0.21, 1.32]
Zidan 2013	18	80	32	80	1.8%	0.44 [0.22, 0.87]
**Total (95% CI)**		**5508**		**6207**	**100.0%**	**0.76 [0.70, 0.82]**
Total events	1953		2583			

Heterogeneity: Chi² = 95.62, df = 36 (P < 0.00001); I² = 62%

Test for overall effect: Z = 6.72 (P < 0.00001)

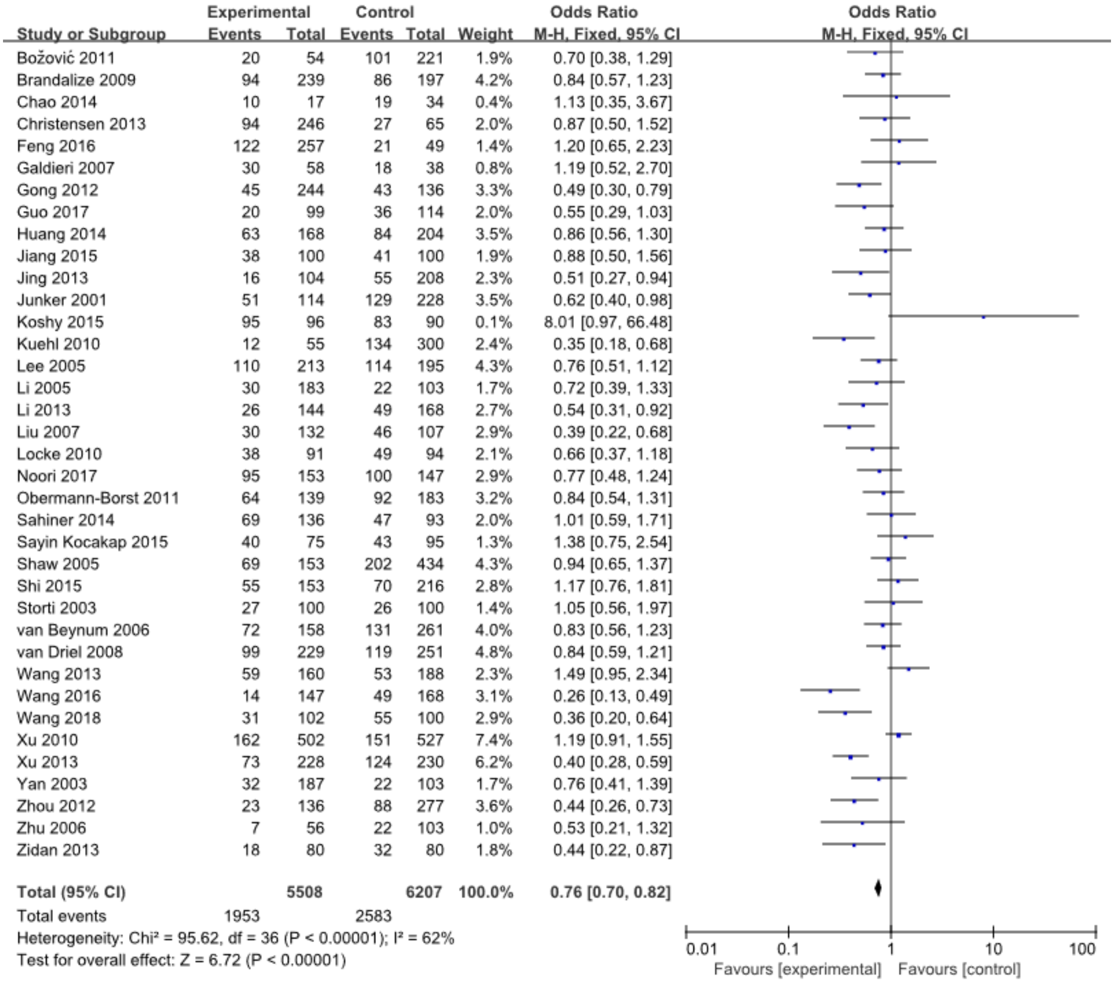

Forest plot of **rs1801133** polymorphism and CHD under dominant comparison

Study or Subgroup	Experimental Events	Experimental Total	Control Events	Control Total	Weight	Odds Ratio M-H, Fixed, 95% CI
Božović 2011	6	54	23	221	1.3%	1.08 [0.42, 2.79]
Brandalize 2009	32	239	18	197	2.9%	1.54 [0.83, 2.83]
Chao 2014	2	17	3	34	0.3%	1.38 [0.21, 9.14]
Christensen 2013	35	246	9	65	2.0%	1.03 [0.47, 2.27]
Feng 2016	21	257	6	49	1.5%	0.64 [0.24, 1.67]
Galdieri 2007	7	58	6	38	1.1%	0.73 [0.23, 2.37]
Gong 2012	76	244	21	136	3.1%	2.48 [1.45, 4.24]
Guo 2017	38	99	30	114	2.9%	1.74 [0.98, 3.12]
Huang 2014	60	168	48	204	4.7%	1.81 [1.15, 2.84]
Jiang 2015	16	100	11	100	1.5%	1.54 [0.68, 3.51]
Jing 2013	46	104	39	208	2.4%	3.44 [2.04, 5.78]
Junker 2001	21	114	21	228	1.9%	2.23 [1.16, 4.27]
Koshy 2015	0	96	0	90		Not estimable
Kuehl 2010	10	55	32	300	1.4%	1.86 [0.86, 4.05]
Lee 2005	14	213	13	195	2.1%	0.98 [0.45, 2.15]
Li 2005	58	183	24	103	3.5%	1.53 [0.88, 2.65]
Li 2013	66	144	35	168	2.9%	3.22 [1.96, 5.28]
Liu 2007	34	132	13	107	1.8%	2.51 [1.25, 5.05]
Locke 2010	14	91	8	94	1.1%	1.95 [0.78, 4.91]
Noori 2017	7	153	1	147	0.2%	7.00 [0.85, 57.61]
Obermann-Borst 2011	9	139	15	183	2.0%	0.78 [0.33, 1.83]
Sahiner 2014	14	136	7	93	1.2%	1.41 [0.55, 3.64]
Sayin Kocakap 2015	2	75	8	95	1.1%	0.30 [0.06, 1.45]
Shaw 2005	16	153	52	434	4.1%	0.86 [0.47, 1.55]
Shi 2015	30	153	45	216	5.0%	0.93 [0.55, 1.55]
Storti 2003	20	100	20	100	2.7%	1.00 [0.50, 2.00]
van Beynum 2006	18	158	23	261	2.6%	1.33 [0.69, 2.55]
van Driel 2008	27	229	25	251	3.5%	1.21 [0.68, 2.15]
Wang 2013	25	160	35	188	4.5%	0.81 [0.46, 1.42]
Wang 2016	60	147	35	168	3.2%	2.62 [1.59, 4.31]
Wang 2018	13	102	3	100	0.4%	4.72 [1.30, 17.12]
Xu 2010	96	502	115	527	15.2%	0.85 [0.63, 1.15]
Xu 2013	49	228	32	230	4.2%	1.69 [1.04, 2.76]
Yan 2003	58	187	24	103	3.6%	1.48 [0.85, 2.57]
Zhou 2012	53	136	63	277	4.2%	2.17 [1.39, 3.38]
Zhu 2006	27	56	24	103	1.5%	3.06 [1.53, 6.14]
Zidan 2013	41	80	27	80	2.2%	2.06 [1.09, 3.91]
**Total (95% CI)**		**5508**		**6207**	**100.0%**	**1.53 [1.38, 1.70]**
Total events	1121		914			

Heterogeneity: Chi² = 85.02, df = 35 (P < 0.00001); I² = 59%
Test for overall effect: Z = 8.22 (P < 0.00001)

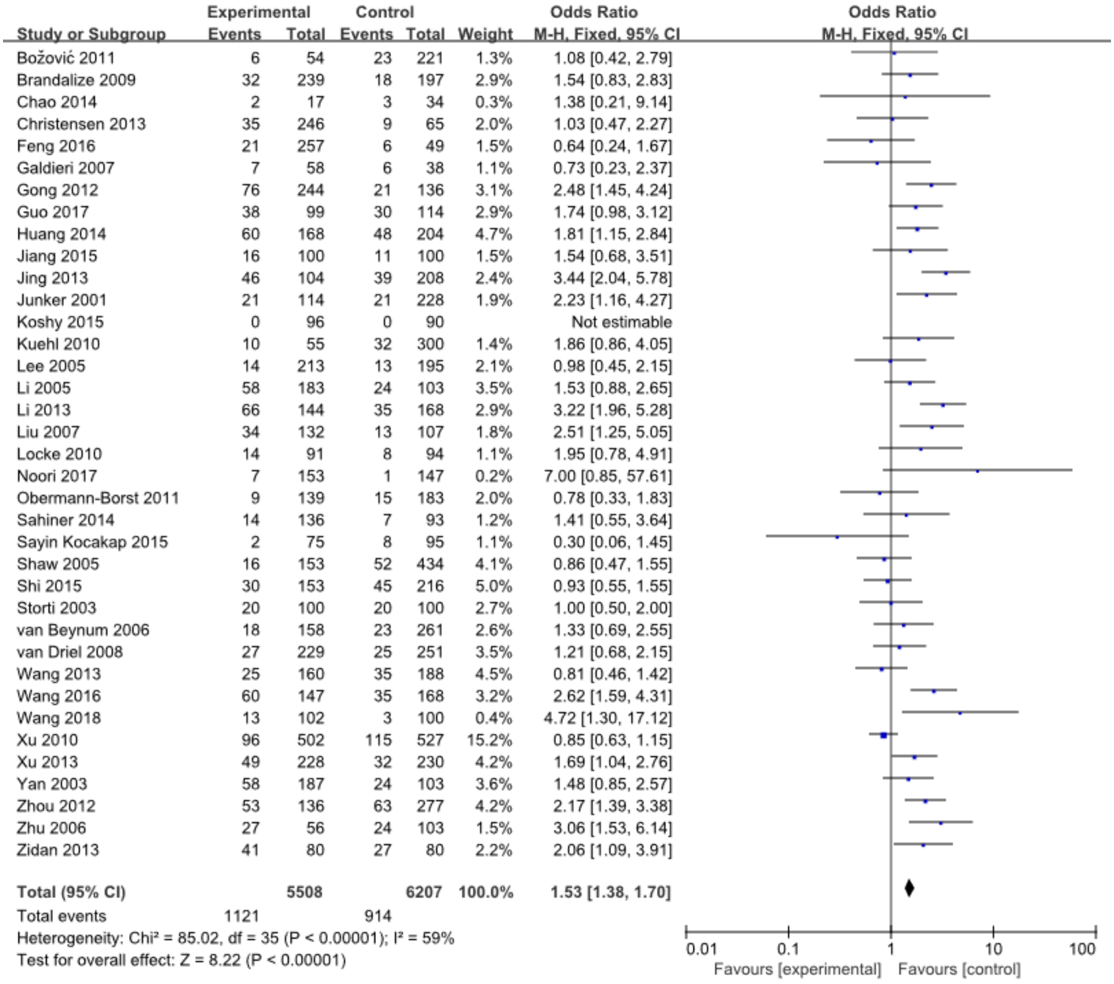

Forest plot of **rs1801133** polymorphism and CHD under recessive comparison

Study or Subgroup	Experimental Events	Experimental Total	Control Events	Control Total	Weight	Odds Ratio M-H, Fixed, 95% CI
Božović 2011	28	54	97	221	1.4%	1.38 [0.76, 2.50]
Brandalize 2009	113	239	93	197	4.2%	1.00 [0.69, 1.46]
Chao 2014	5	17	12	34	0.4%	0.76 [0.22, 2.69]
Christensen 2013	117	246	29	65	1.9%	1.13 [0.65, 1.95]
Feng 2016	114	257	22	49	1.6%	0.98 [0.53, 1.81]
Galdieri 2007	21	58	14	38	0.8%	0.97 [0.42, 2.27]
Gong 2012	123	244	72	136	3.5%	0.90 [0.59, 1.38]
Guo 2017	41	99	48	114	2.0%	0.97 [0.56, 1.68]
Huang 2014	45	168	72	204	3.7%	0.67 [0.43, 1.05]
Jiang 2015	46	100	48	100	2.0%	0.92 [0.53, 1.61]
Jing 2013	42	104	114	208	3.5%	0.56 [0.35, 0.90]
Junker 2001	42	114	78	228	2.5%	1.12 [0.70, 1.79]
Koshy 2015	1	96	7	90	0.6%	0.12 [0.02, 1.04]
Kuehl 2010	33	55	134	300	1.3%	1.86 [1.03, 3.34]
Lee 2005	89	213	68	195	3.2%	1.34 [0.90, 2.00]
Li 2005	95	183	57	103	2.7%	0.87 [0.54, 1.41]
Li 2013	52	144	84	168	3.8%	0.57 [0.36, 0.89]
Liu 2007	68	132	48	107	2.0%	1.31 [0.78, 2.18]
Locke 2010	39	91	37	94	1.6%	1.16 [0.64, 2.08]
Noori 2017	51	153	46	147	2.4%	1.10 [0.68, 1.78]
Obermann-Borst 2011	66	139	76	183	2.7%	1.27 [0.82, 1.98]
Sahiner 2014	53	136	39	93	2.2%	0.88 [0.52, 1.51]
Sayin Kocakap 2015	33	75	44	95	1.7%	0.91 [0.50, 1.67]
Shaw 2005	68	153	180	434	4.0%	1.13 [0.78, 1.64]
Shi 2015	68	153	101	216	3.6%	0.91 [0.60, 1.38]
Storti 2003	53	100	54	100	2.0%	0.96 [0.55, 1.67]
van Beynum 2006	68	158	107	261	3.6%	1.09 [0.73, 1.62]
van Driel 2008	103	229	107	251	4.3%	1.10 [0.77, 1.58]
Wang 2013	76	160	100	188	3.7%	0.80 [0.52, 1.21]
Wang 2016	73	147	84	168	3.1%	0.99 [0.63, 1.54]
Wang 2018	58	102	42	100	1.4%	1.82 [1.04, 3.18]
Xu 2010	244	502	261	527	10.1%	0.96 [0.75, 1.23]
Xu 2013	106	228	74	230	3.0%	1.83 [1.25, 2.68]
Yan 2003	97	187	57	103	2.7%	0.87 [0.54, 1.41]
Zhou 2012	60	136	126	277	3.6%	0.95 [0.63, 1.43]
Zhu 2006	22	56	57	103	1.9%	0.52 [0.27, 1.01]
Zidan 2013	21	80	21	80	1.2%	1.00 [0.49, 2.02]
**Total (95% CI)**		**5508**		**6207**	**100.0%**	**1.01 [0.93, 1.09]**
Total events	2434		2710			

Heterogeneity: $Chi^2 = 50.56$, df = 36 (P = 0.05); $I^2 = 29\%$
Test for overall effect: Z = 0.17 (P = 0.86)

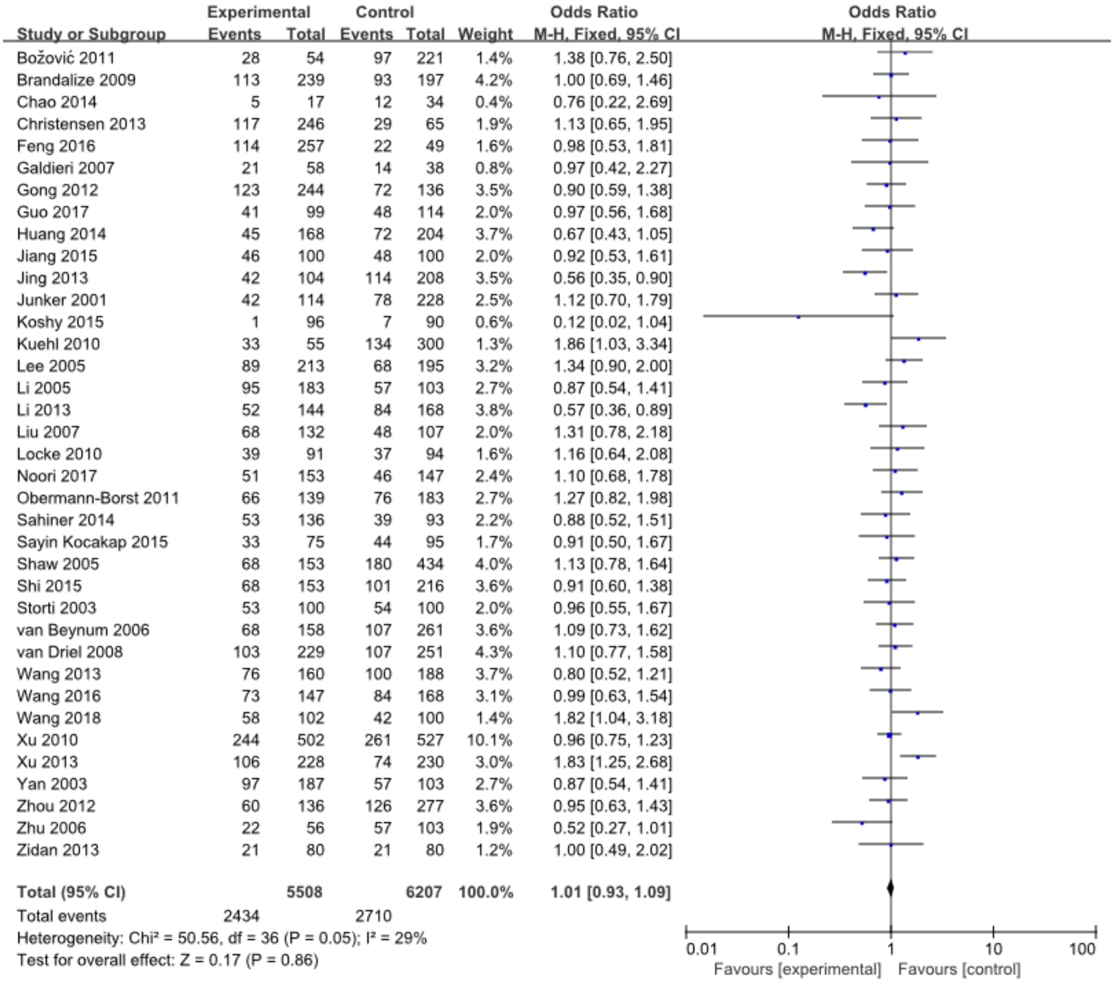

Forest plot of **rs1801133** polymorphism and CHD under additive comparison

Study or Subgroup	Experimental Events	Experimental Total	Control Events	Control Total	Weight	Odds Ratio M-H, Fixed, 95% CI
Božović 2011	68	108	299	442	1.6%	0.81 [0.52, 1.26]
Brandalize 2009	301	478	265	394	3.8%	0.83 [0.63, 1.10]
Chao 2014	25	34	50	68	0.3%	1.00 [0.39, 2.54]
Christensen 2013	305	492	83	130	1.8%	0.92 [0.62, 1.38]
Feng 2016	358	514	64	98	1.2%	1.22 [0.77, 1.92]
Galdieri 2007	81	116	50	76	0.7%	1.20 [0.65, 2.23]
Gong 2012	213	488	158	272	4.1%	0.56 [0.41, 0.75]
Guo 2017	81	198	120	228	2.4%	0.62 [0.42, 0.92]
Huang 2014	171	336	240	408	3.8%	0.73 [0.54, 0.97]
Jiang 2015	122	200	130	200	1.8%	0.84 [0.56, 1.26]
Jing 2013	74	208	224	416	3.4%	0.47 [0.34, 0.67]
Junker 2001	144	228	336	456	3.0%	0.61 [0.44, 0.86]
Koshy 2015	191	192	173	180	0.0%	7.73 [0.94, 63.45]
Kuehl 2010	57	110	402	600	2.1%	0.53 [0.35, 0.80]
Lee 2005	309	426	296	390	3.0%	0.84 [0.61, 1.15]
Li 2005	155	366	101	206	2.7%	0.76 [0.54, 1.08]
Li 2013	104	288	182	336	3.8%	0.48 [0.35, 0.66]
Liu 2007	128	264	140	214	2.8%	0.50 [0.34, 0.72]
Locke 2010	115	182	135	188	1.7%	0.67 [0.43, 1.04]
Noori 2017	241	306	246	294	1.9%	0.72 [0.48, 1.09]
Obermann-Borst 2011	194	278	260	366	2.4%	0.94 [0.67, 1.32]
Sahiner 2014	191	272	133	186	1.7%	0.94 [0.62, 1.42]
Sayin Kocakap 2015	113	150	130	190	1.0%	1.41 [0.87, 2.28]
Shaw 2005	206	306	584	868	3.6%	1.00 [0.76, 1.32]
Shi 2015	178	306	241	432	3.0%	1.10 [0.82, 1.48]
Storti 2003	107	200	106	200	1.8%	1.02 [0.69, 1.51]
van Beynum 2006	212	316	369	522	3.3%	0.85 [0.63, 1.14]
van Driel 2008	301	458	345	502	4.0%	0.87 [0.67, 1.14]
Wang 2013	194	320	206	376	2.7%	1.27 [0.94, 1.72]
Wang 2016	101	294	182	336	4.0%	0.44 [0.32, 0.61]
Wang 2018	120	204	152	200	2.3%	0.45 [0.29, 0.69]
Xu 2010	568	1004	563	1054	8.5%	1.14 [0.95, 1.35]
Xu 2013	252	456	322	460	5.1%	0.53 [0.40, 0.69]
Yan 2003	161	374	101	206	2.7%	0.79 [0.56, 1.11]
Zhou 2012	106	272	302	554	4.3%	0.53 [0.40, 0.72]
Zhu 2006	36	112	101	206	1.7%	0.49 [0.30, 0.80]
Zidan 2013	57	160	85	160	2.0%	0.49 [0.31, 0.76]
**Total (95% CI)**		**11016**		**12414**	**100.0%**	**0.77 [0.73, 0.81]**
Total events	6340		7876			

Heterogeneity: $Chi^2 = 133.07$, df = 36 ($P < 0.00001$); $I^2 = 73\%$

Test for overall effect: $Z = 9.20$ ($P < 0.00001$)

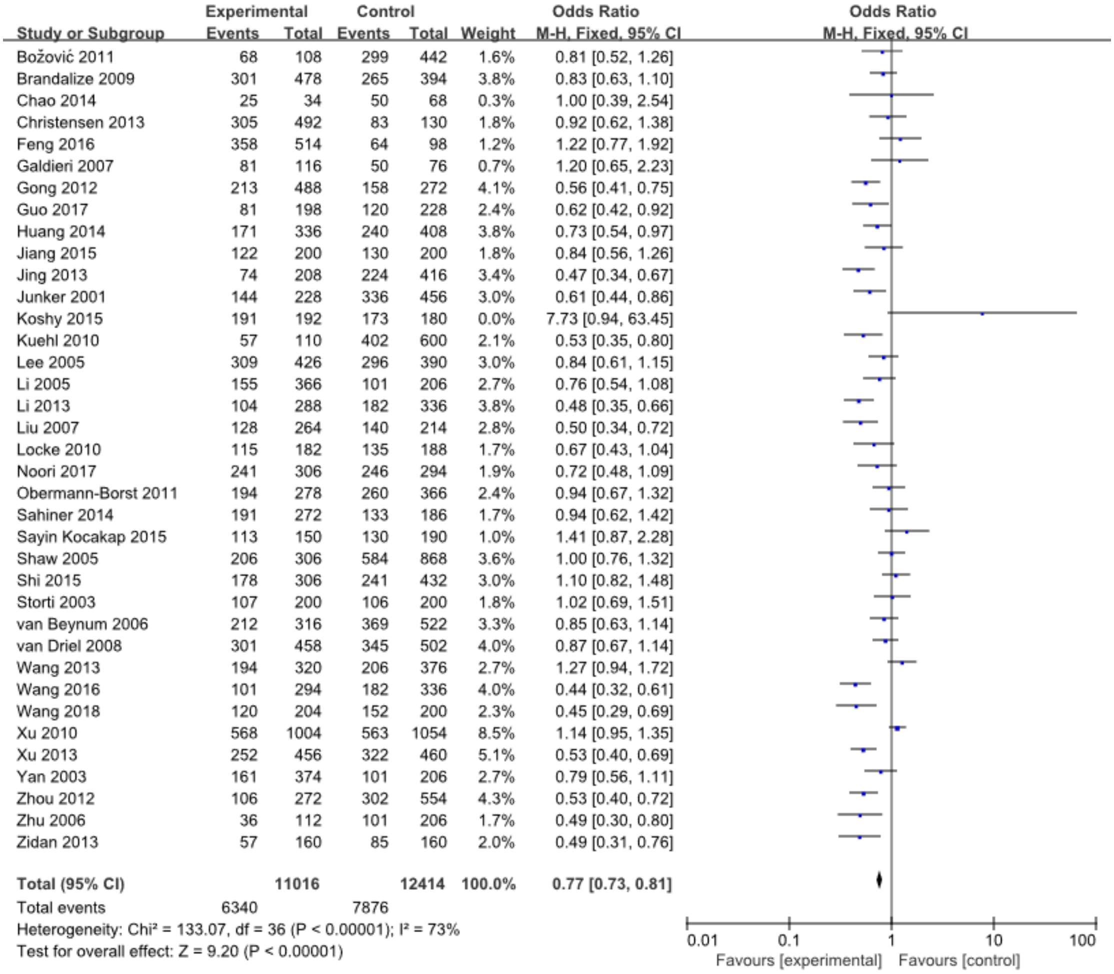

Forest plot of **rs1801133** polymorphism and CHD under allele comparison

Funnel plots of investigated polymorphisms

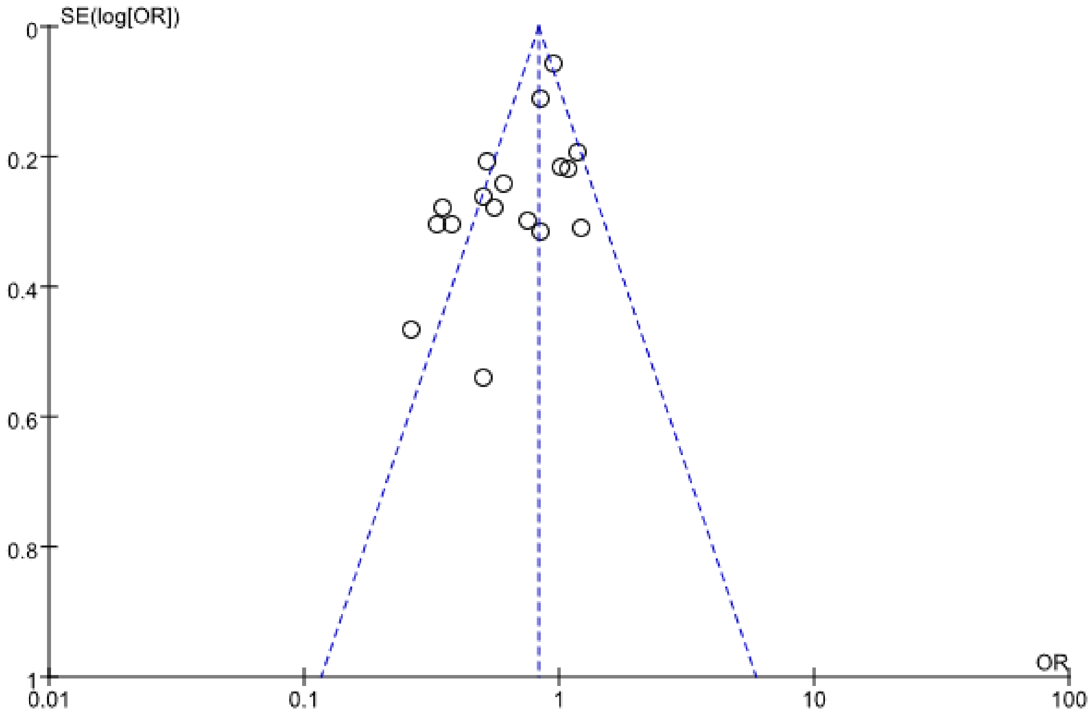

Funnel plot of **rs1801394** polymorphism and CHD under dominant comparison

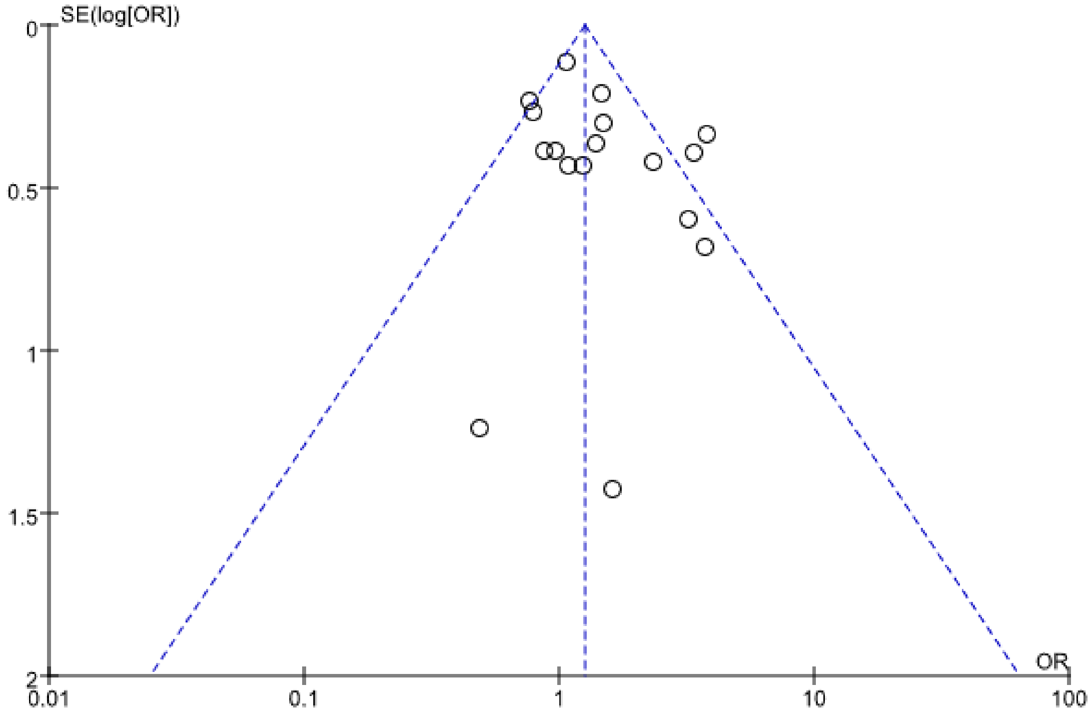

Funnel plot of **rs1801394** polymorphism and CHD under recessive comparison

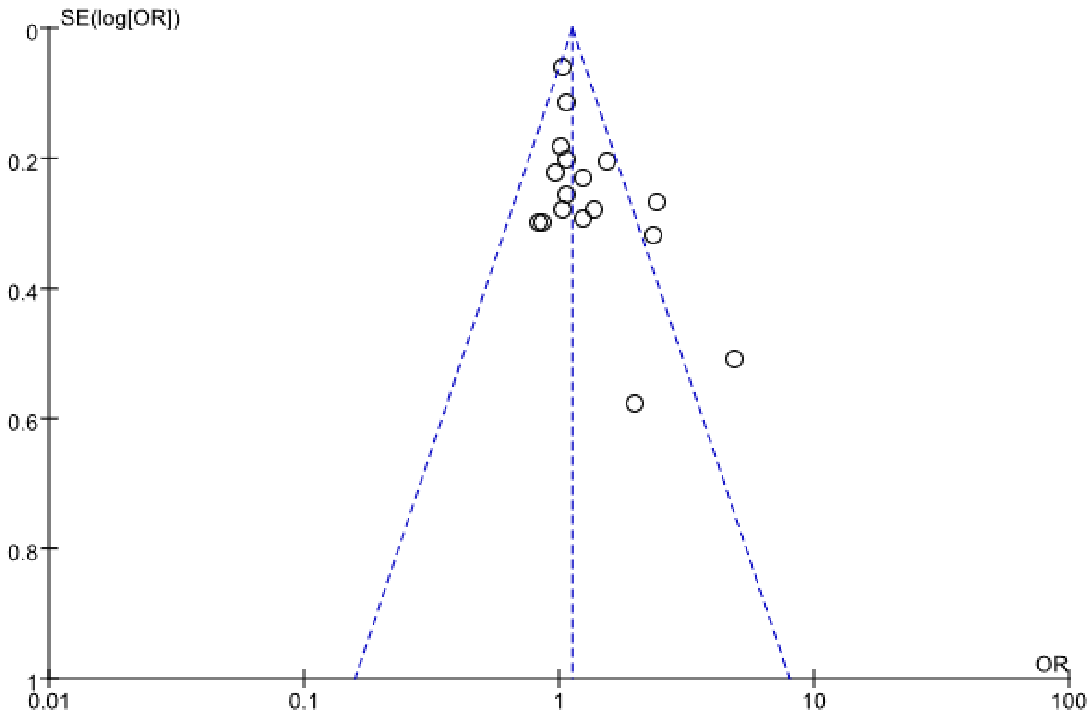

Funnel plot of **rs1801394** polymorphism and CHD under additive comparison

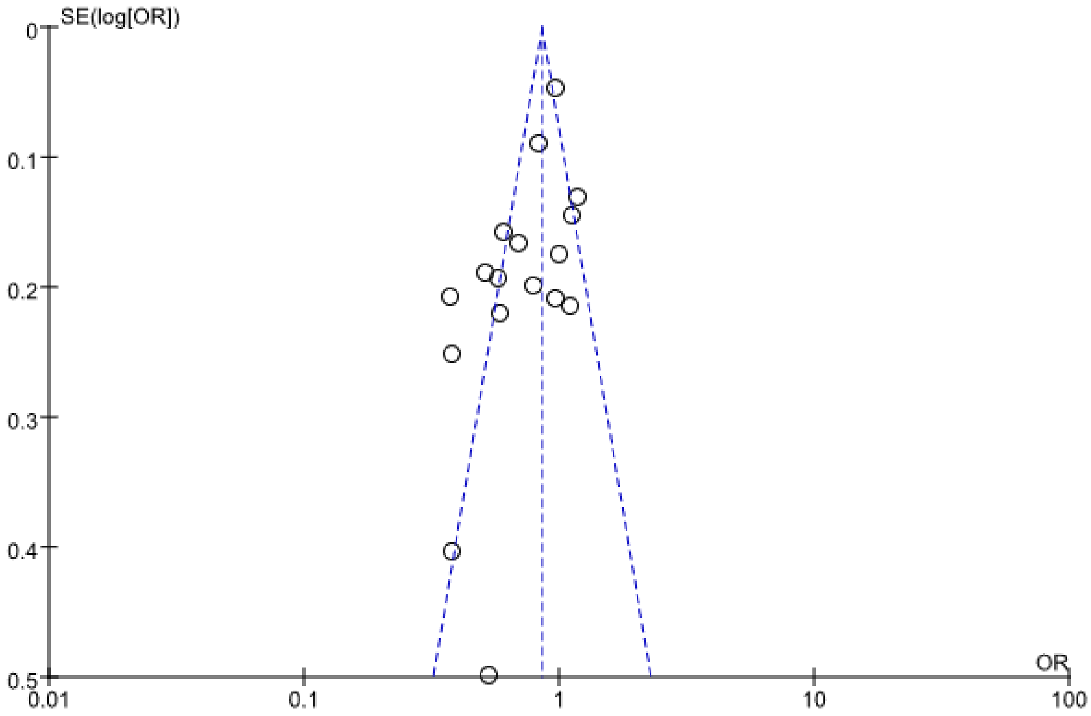

Funnel plot of **rs1801394** polymorphism and CHD under allele comparison

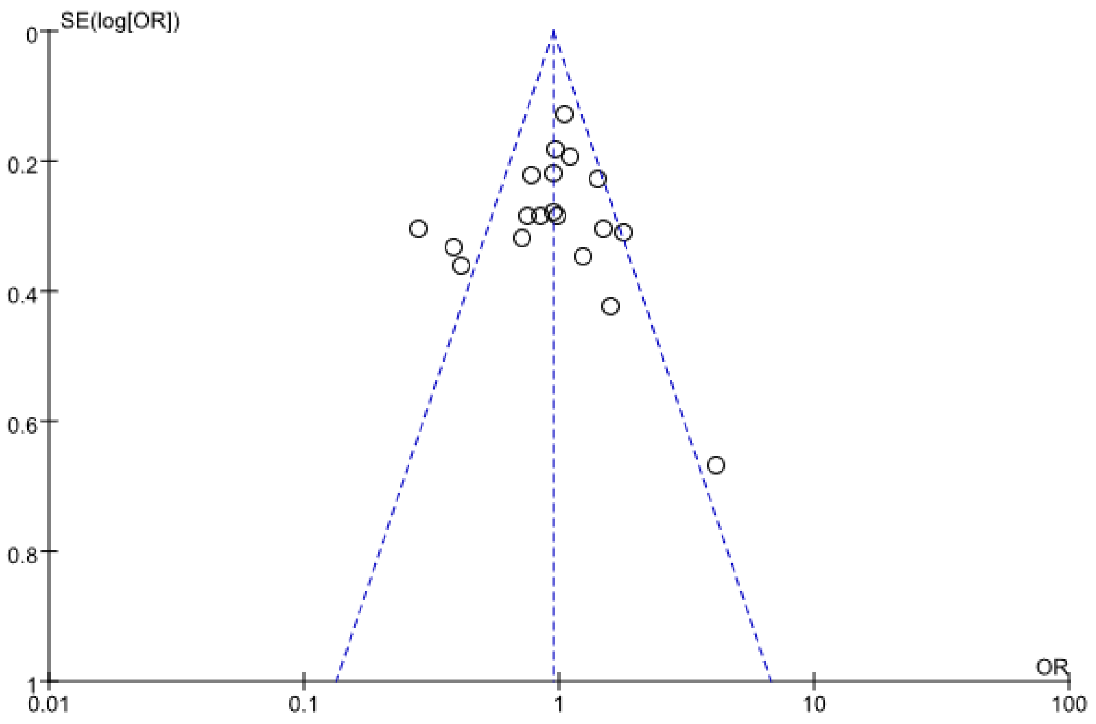

Funnel plot of **rs1801131** polymorphism and CHD under dominant comparison

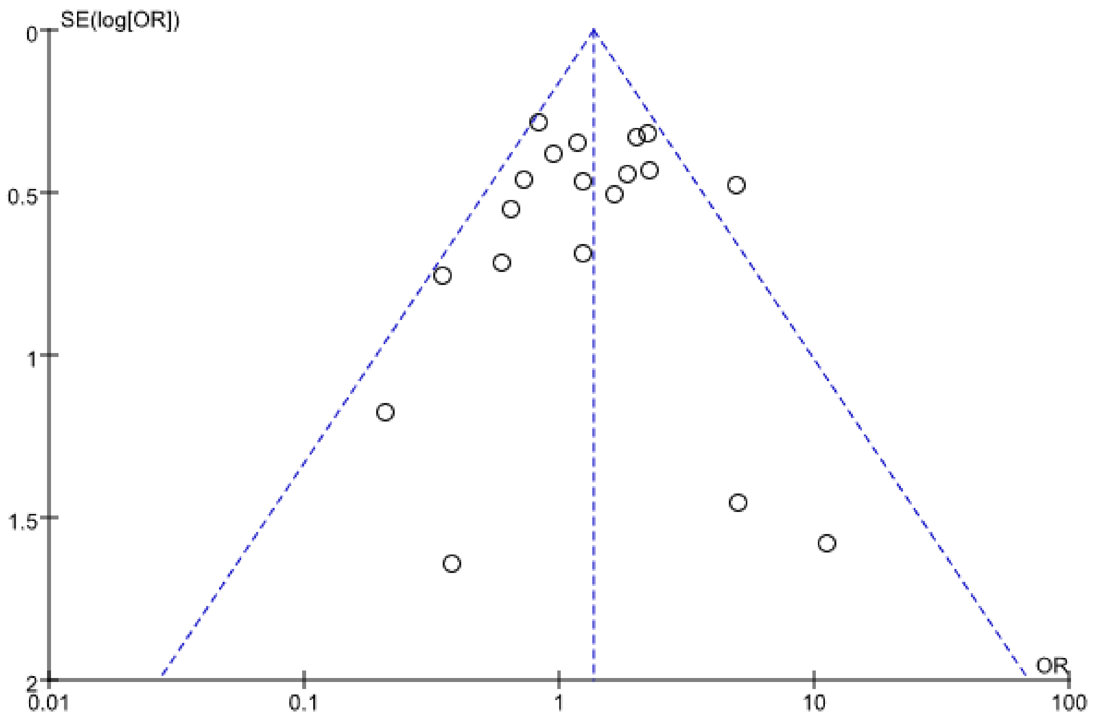

Funnel plot of **rs1801131** polymorphism and CHD under recessive comparison

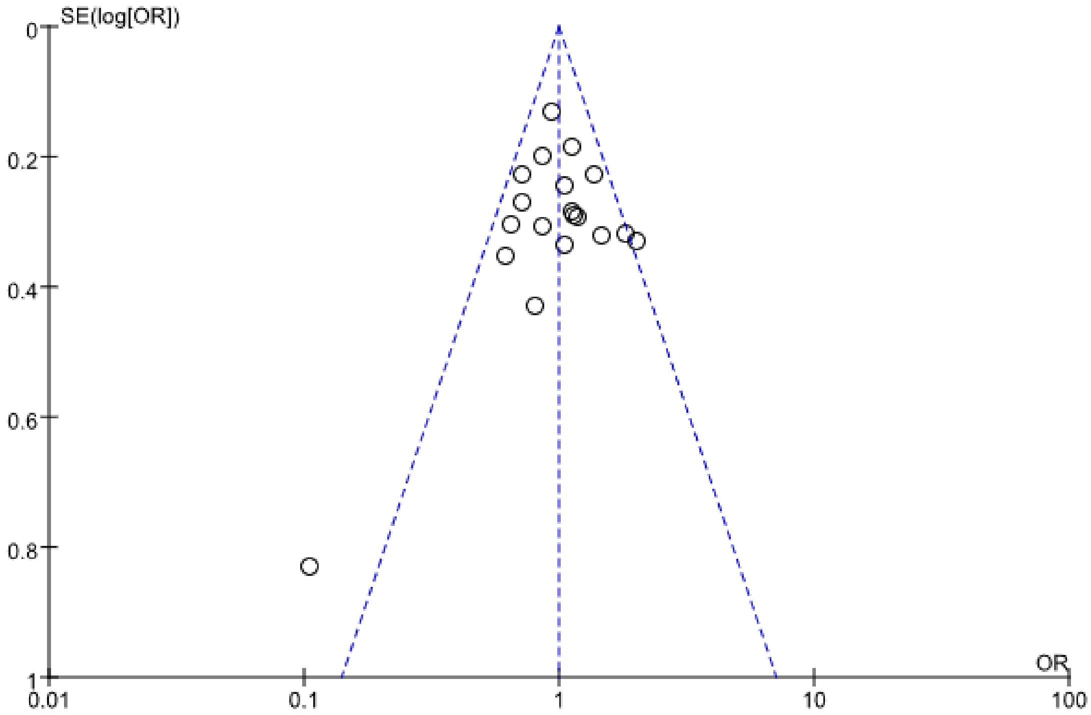

Funnel plot of **rs1801131** polymorphism and CHD under additive comparison

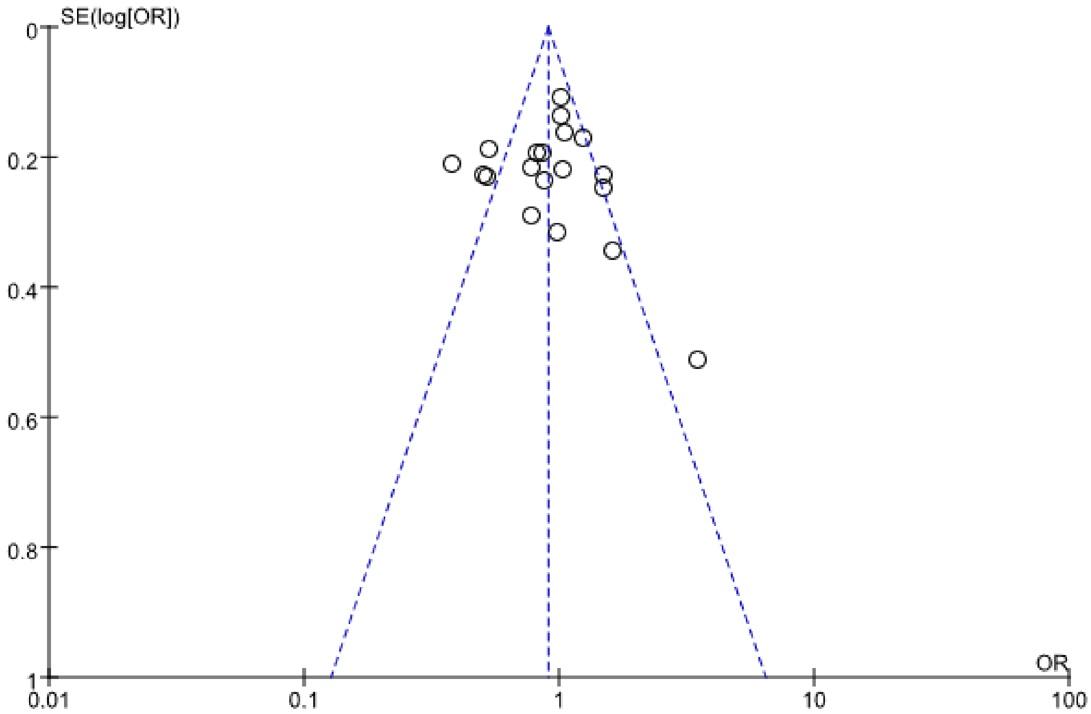

Funnel plot of **rs1801131** polymorphism and CHD under allele comparison

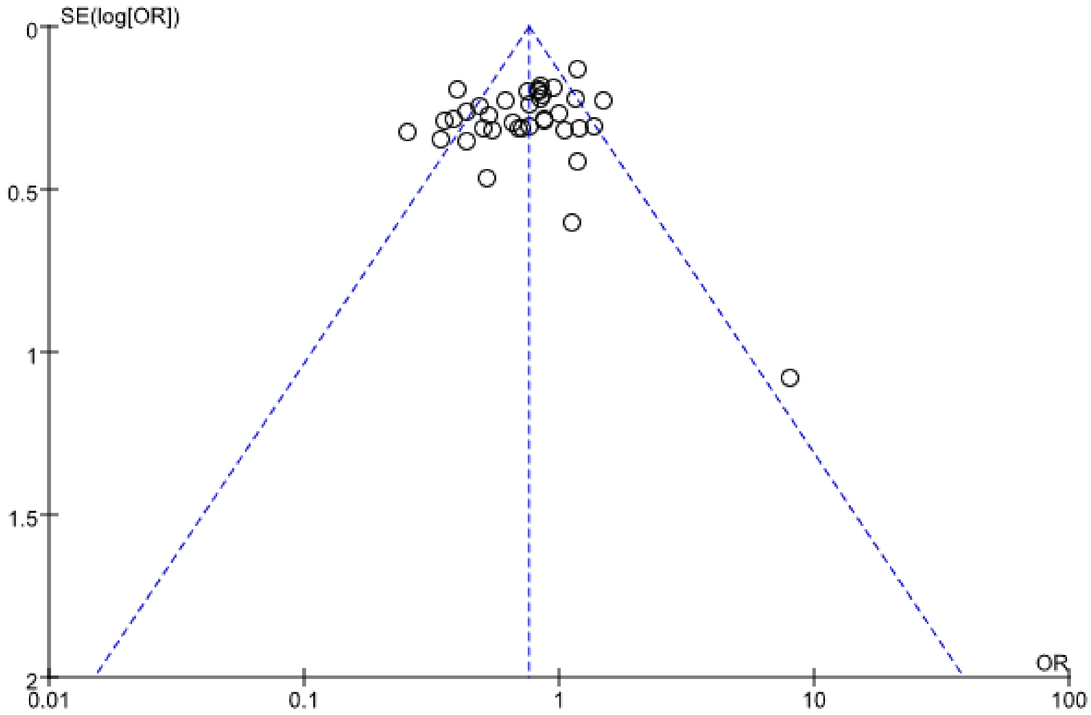

Funnel plot of **rs1801133** polymorphism and CHD under dominant comparison

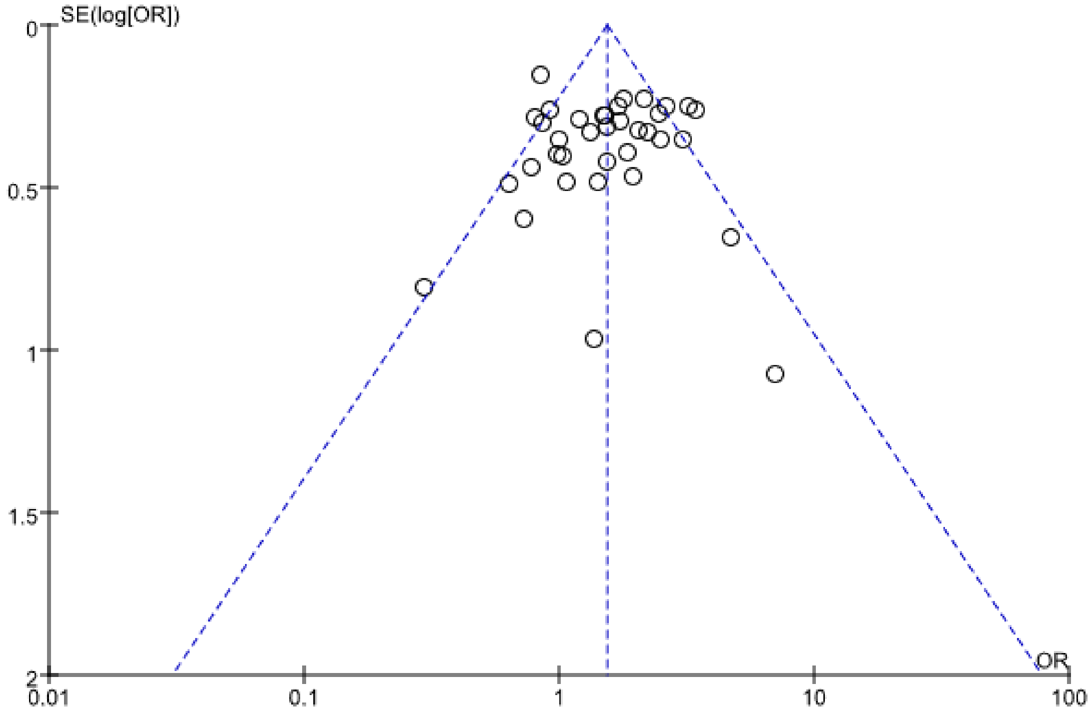

Funnel plot of **rs1801133** polymorphism and CHD under recessive comparison

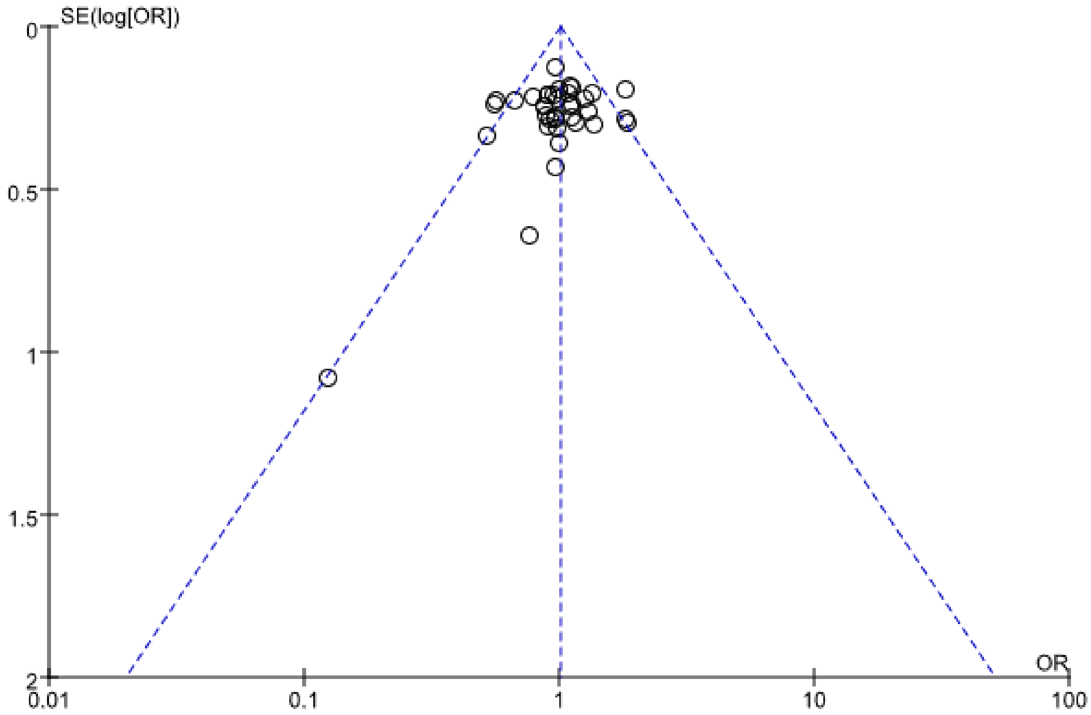

Funnel plot of **rs1801133** polymorphism and CHD under additive comparison

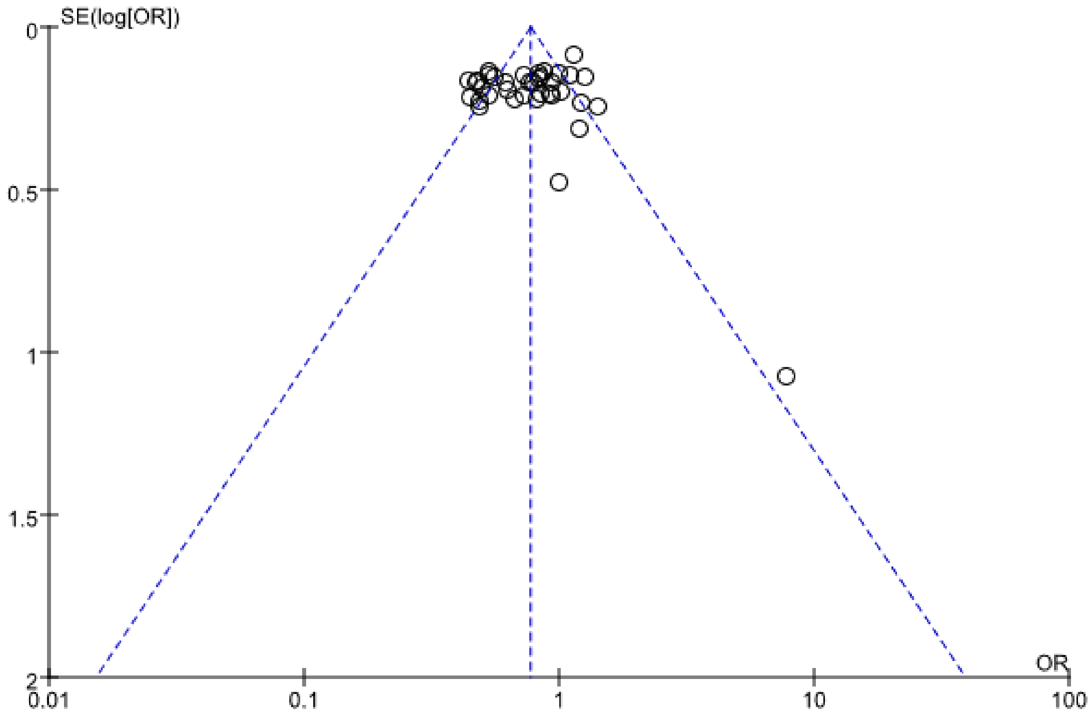

Funnel plot of **rs1801133** polymorphism and CHD under allele comparison
